# The Kinetochore Receptor for the Cohesin Loading Complex

**DOI:** 10.1016/j.cell.2017.08.017

**Published:** 2017-09-21

**Authors:** Stephen M. Hinshaw, Vasso Makrantoni, Stephen C. Harrison, Adèle L. Marston

**Affiliations:** 1 Department of Biological Chemistry and Molecular Pharmacology, Harvard Medical School, Boston, MA 02115, USA; 2 The Wellcome Trust Centre for Cell Biology, School of Biological Sciences, University of Edinburgh, Edinburgh EH9 3BF, UK; 3 Howard Hughes Medical Institute, Chevy Chase, MD, USA

**Keywords:** centromere, cohesin, kinetochore, cell cycle

## Abstract

The ring-shaped cohesin complex brings together distant DNA domains to maintain, express, and segregate the genome. Establishing specific chromosomal linkages depends on cohesin recruitment to defined loci. One such locus is the budding yeast centromere, which is a paradigm for targeted cohesin loading. The kinetochore, a multiprotein complex that connects centromeres to microtubules, drives the recruitment of high levels of cohesin to link sister chromatids together. We have exploited this system to determine the mechanism of specific cohesin recruitment. We show that phosphorylation of the Ctf19 kinetochore protein by a conserved kinase, DDK, provides a binding site for the Scc2/4 cohesin loading complex, thereby directing cohesin loading to centromeres. A similar mechanism targets cohesin to chromosomes in vertebrates. These findings represent a complete molecular description of targeted cohesin loading, a phenomenon with wide-ranging importance in chromosome segregation and, in multicellular organisms, transcription regulation.

## Highlights

• Reconstitution of a phosphorylation-dependent pathway for targeted cohesin loading
• The mechanism by which kinetochores enhance sister centromere cohesion

## Graphical Abstract

**Figure:**
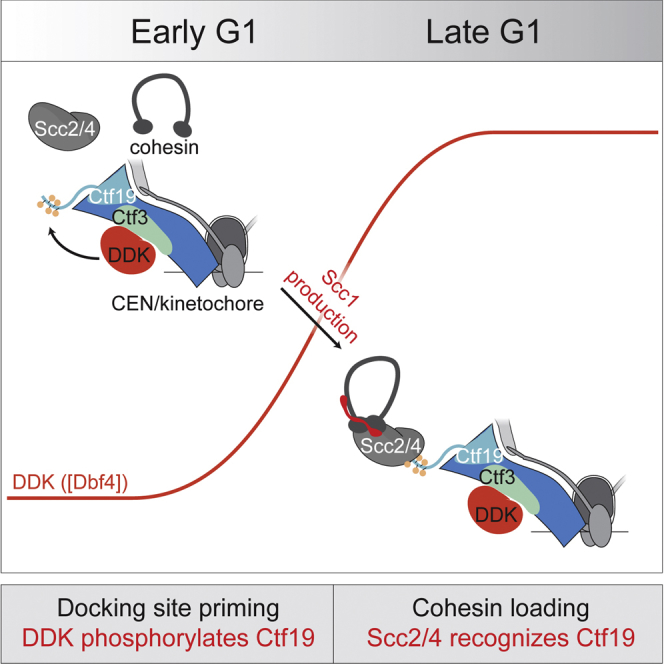

## Teaser

The mechanism by which cohesin is recruited and loaded in yeast and vertebrates is elucidated with implications for our understanding of chromosome segregation.

## Introduction

Genome maintenance, expression, and transmission depend on organization of chromosomes into functional domains. At the heart of this organization, in both prokaryotes and eukaryotes, are protein complexes in the structural maintenance of chromosomes (SMC) family: cohesin, condensin, and SMC5/6 (Uhlmann, 2016). These complexes must be targeted to specific regions of the chromosome, including areas of damage, transcriptional units, and centromeres. Biological control of SMC complex activity thus relies on interactions that direct these complexes to particular chromosomal sites at appropriate times.

The best understood of the SMC complexes is cohesin, which links newly duplicated chromosomes in preparation for their segregation during cell division. At the core of the cohesin complex is a three-protein ring (Smc1, Smc3, and Scc1) (Losada and Hirano, 2005, Nasmyth, 2011, Onn et al., 2008). A separate and broadly conserved two-protein complex called Scc2/4 in yeast and NIPBL/Mau2 in humans loads cohesin onto chromosomes (Ciosk et al., 2000). While the yeast Scc2 and Scc4 genes were cloned initially due to their role in sister chromatid cohesion, the fly homolog of Scc2, Nipped-B, was first identified as a gene required for proper enhancer function (Rollins et al., 1999). Heterozygous mutations in NIPBL cause Cornelia de Lange syndrome (CdLS), a developmental disorder associated with aberrant transcription across the genome (Kawauchi et al., 2009, Krantz et al., 2004, Tonkin et al., 2004). The dual role of cohesin loading in chromosome segregation and transcription regulation (Fay et al., 2011) makes determining molecular pathways for targeted cohesin loading an issue of broad interest.

Genomic locations of cohesin enrichment exist in all eukaryotes, but the phenomenon is best understood in the context of the yeast centromere. Cohesin density peaks at or near centromeres and extends to nearby pericentromeric regions (Glynn et al., 2004, Lengronne et al., 2004). In mitosis, pericentromeric cohesin complexes enable spindle assembly checkpoint silencing by resisting the poleward forces exerted by kinetochore microtubules (Stern and Murray, 2001, Tanaka et al., 2000). Disabling this process causes high rates of chromosome missegregation and aneuploidy, both pervasive features of cancer (Holland and Cleveland, 2012). In meiosis, high pericentromeric cohesin occupancy serves at least three functions critical for avoiding aneuploidy (Nasmyth, 2001). First, at least in fission yeast, it prevents biorientation of sister kinetochores in meiosis I, thus facilitating reductional chromosome segregation (Sakuno et al., 2009, Watanabe and Nurse, 1999). Second, its selective preservation at centromeres throughout meiosis I is required for accurate chromosome segregation in meiosis II (Lee and Orr-Weaver, 2001). Third, it represses crossover recombination close to centromeres (Vincenten et al., 2015), which would otherwise lead to meiosis II nondisjunction (Rockmill et al., 2006).

Preferential cohesin loading at centromeres is a kinetochore-dependent process (Megee and Koshland, 1999, Tanaka et al., 2000, Weber et al., 2004). The yeast Ctf19 complex, the homolog of the human Constitutive Centromere Associated Network (CCAN), enables this function by recruiting the cohesin loader to centromeres (Eckert et al., 2007, Fernius and Marston, 2009, Fernius et al., 2013, Kogut et al., 2009, Ng et al., 2009). We have shown previously that Scc2 recruitment to centromeres requires a conserved surface on the Scc4 protein (Hinshaw et al., 2015). Mutation of amino acid residues at this surface abrogates Scc2/4 recruitment to centromeres, causes loss of cohesion specifically at centromeres, and leads to elevated rates of minichromosome missegregation. In the work reported here, we sought to identify the kinetochore factor mediating Scc2/4 recruitment, to describe the mode of regulation that restricts specific centromeric cohesin loading to a narrow window of the cell cycle, and to understand whether a similar recruitment process operates in other species.

One factor likely to play a broad role in regulating targeted cohesin loading is the Dbf4-dependent kinase (DDK). DDK is a cell-cycle-regulated kinase that is required for the preferential association of cohesin with centromeres in budding and fission yeast and with unreplicated chromatin in vertebrates (Bailis et al., 2003, Natsume et al., 2013, Takahashi et al., 2008). The essential function of DDK is to activate the Mcm2-7 helicase for DNA replication (Sheu and Stillman, 2010). In yeast, the Ctf19 complex recruits DDK to kinetochores in G1, where its activity enables subsequent Scc2 recruitment and robust centromere cohesion independently of its role in DNA replication (Natsume et al., 2013). In *Xenopus laevis* egg extracts, DDK activity is required for the association of Scc2/4 with replication origins (Takahashi et al., 2008). These findings suggest that DDK is part of a common mechanism for directing cohesin loading activity to defined chromosomal locations and for coupling this activity with the onset of DNA replication.

Using the budding yeast centromere as a model, we have determined the molecular pathway that drives targeted cohesin loading. We report here that DDK phosphorylates the Ctf19 protein. Phosphorylated Ctf19 interacts directly with the previously identified conserved surface of Scc4, explaining both the requirement for DDK in centromeric cohesion establishment and the nature of Scc4-mediated targeting of cohesin loading to centromeres. Our results thus provide a full description of the molecular events required to recruit cohesin to a defined chromosomal locus.

## Results

### DDK Phosphorylates Ctf19-Mcm21

**Figure 1:**
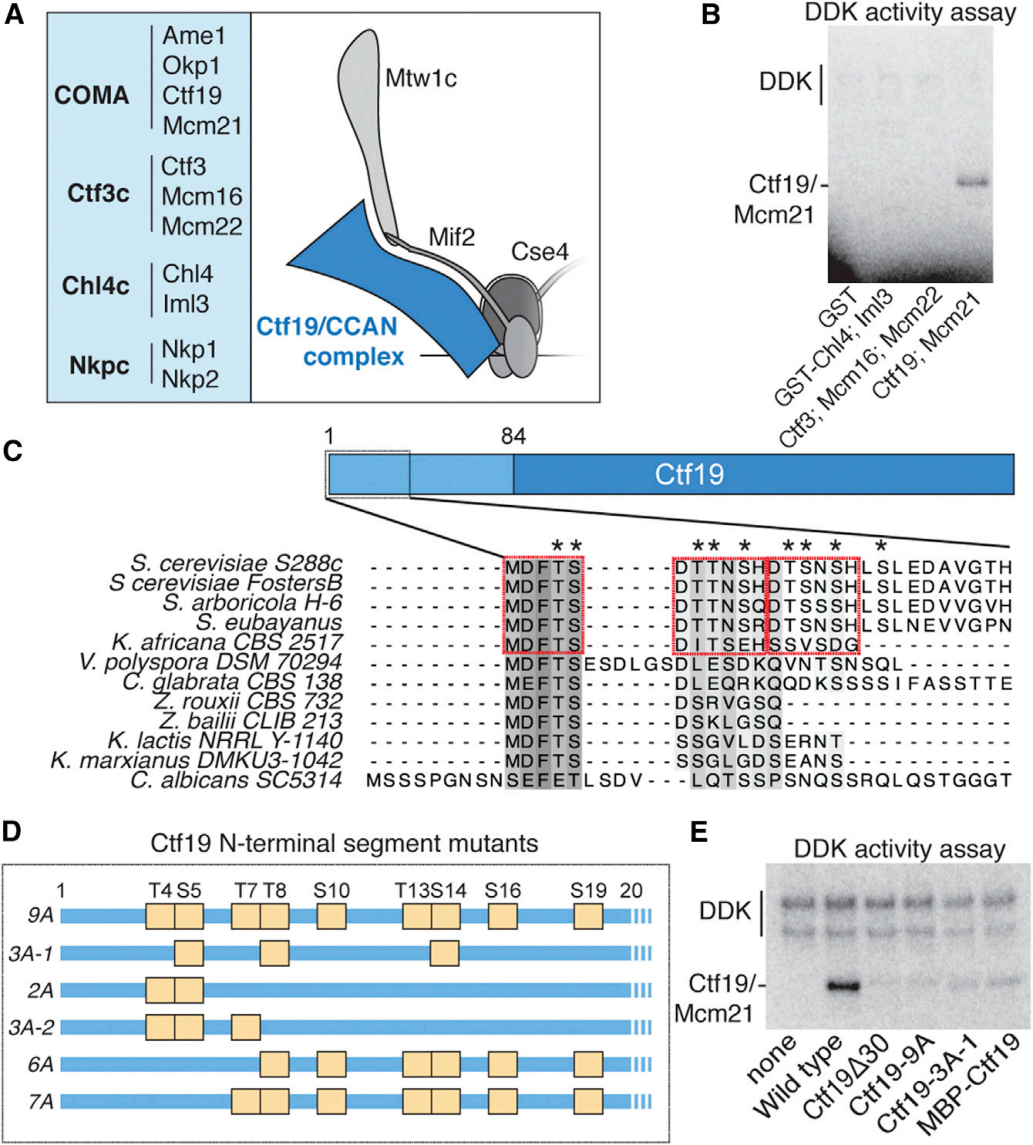
DDK Phosphorylates Ctf19 (A) Ctf19 subcomplexes and their members (left) listed next to a schematic of the kinetochore with the Ctf19 complex shown in blue (right). (B) In vitro phosphorylation of the Ctf19-Mcm21 dimer by DDK. The indicated substrate proteins were incubated with purified DDK and γ-^32^[P]-ATP. Reaction products were resolved by SDS-PAGE and visualized by autoradiography. (C) Diagram of Ctf19 showing the fragment resolved crystallographically (dark blue, PDB: 3ZXU) and the unstructured N-terminal region (light blue). Sequence alignment shows the N-terminal fragment. Red boxes indicate candidate phosphorylation motifs. Asterisks mark residues mutated in the *ctf19*-*9A* allele. (D) Ctf19 alleles used in this work. Yellow boxes indicate residues mutated to alanine. (E) DDK phosphorylates residues in the Ctf19 N-terminal region. Kinase assay performed as in (B). Substrates are indicated below. See also Figures S1–S3 and Table S1.

**Figure S1:**
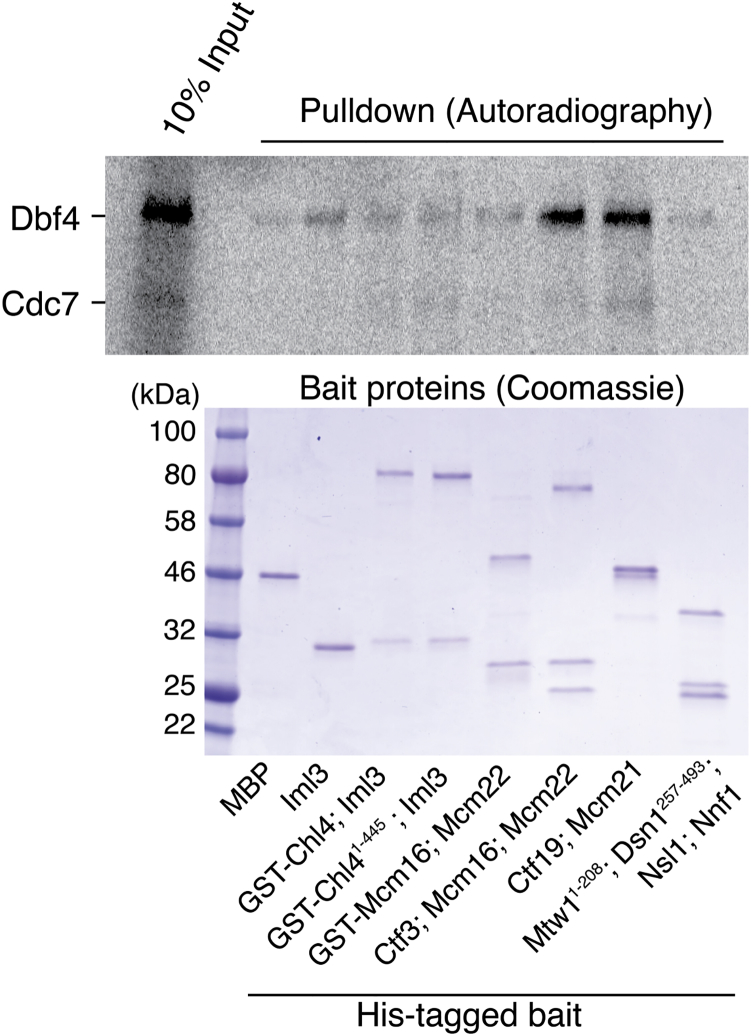
Reconstitution of DDK Association with Ctf19 Complex Proteins, Related to Figure 1 Dbf4 and Cdc7 were translated in vitro in the presence of ^35^S-labeled methionine and incubated together with the indicated 6His-tagged Ctf19 complex members. Bead-bound proteins after Ni^2+^-affinity pulldowns were analyzed by SDS-PAGE and autoradiography (top). Purified bait proteins were analyzed separately by Coomassie stain (bottom). DDK associates with the Ctf3 trimer and with the Ctf19-Mcm21 dimer.

Several observations suggest that DDK phosphorylates a component of the Ctf19 complex (Figure 1A) and that this signal recruits Scc2 to centromeres. First, DDK localization at kinetochores immediately precedes Scc2 localization and subsequent cohesin loading (Natsume et al., 2013). Second, Scc4 has a conserved, positively charged, surface patch important for Scc2 localization, leading to the suggestion that this patch might be a phosphopeptide binding motif (Chao et al., 2015, Hinshaw et al., 2015). Third, the interaction between Scc2/4 and DDK is probably indirect in yeast, as it is not observable in a reconstituted pulldown system (our unpublished observations). To determine whether DDK can bind proteins of the Ctf19 complex, we performed pulldowns using recombinant Ctf19 complex proteins and detected associated radiolabeled DDK produced by in vitro translation (Figure S1). We identified two candidate DDK interaction partners: the Ctf19-Mcm21 dimer and the Ctf3 complex. Although the Ctf19-Mcm21 dimer associates with the Okp1 and Ame1 proteins to make the COMA tetramer (CENP-P/O/Q/U in humans) (De Wulf et al., 2003, Okada et al., 2006), Okp1 and Ame1 were not required for DDK interaction. The Ctf3 complex is a trimeric assembly of Ctf3, Mcm16, and Mcm22 (CENP-I/H/K in humans) (Measday et al., 2002, Okada et al., 2006), and Ctf3 was the component required for DDK interaction.

**Figure S2:**
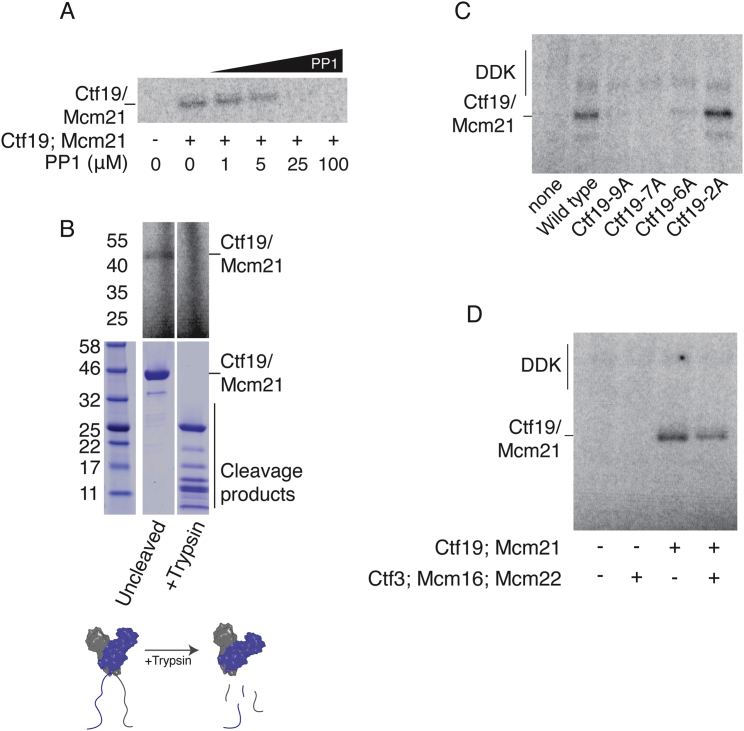
Determinants of Ctf19 Phosphorylation by DDK, Related to Figure 1 (A) Products of phosphorylation reactions containing Ctf19-Mcm21, analog-sensitive DDK, γ-^32^[P]-ATP, and increasing concentrations of PP1. (B) Reactions carried out as in panel A, except wild-type DDK was used as in Figure 1B. After phosphorylation, reactions were subjected to a brief cleavage reaction by incubation with trypsin (top, autoradiography). Identical cleavage reactions were carried out in the absence of DDK and ATP to identify Ctf19-Mcm21 cleavage fragments (bottom; Coomassie). A schematic of the cleavage reaction is shown below (PDB: 3ZXU). (C) Phosphorylation of mutant Ctf19-Mcm21 complexes by purified DDK. Phosphorylation reactions were carried out as in panel A except that PP1 was not included. Substrates are indicated below. (D) The Ctf3 complex does not enhance Ctf19-Mcm21 phosphorylation in vitro. Reactions were carried out as in (B) in the presence or absence of an equimolar amount of purified Ctf3 complex.

The identification of Ctf19-Mcm21 and Ctf3 as DDK binding partners suggested that they could be the kinetochore substrates that generate the signal for recruitment of Scc2 to centromeres. We therefore purified DDK from yeast and performed kinase activity assays using Ctf19 complex proteins as substrates. Purified DDK phosphorylated Ctf19-Mcm21 but not the Ctf3 trimer or the Chl4-Iml3 dimer (Figure 1B). To confirm that phosphate transfer to Ctf19-Mcm21 depended on the catalytic activity of Cdc7 and not a co-purifying kinase, we purified an ATP analog (PP1)-sensitive mutant of DDK (Wan et al., 2006). Phosphate transfer to Ctf19 was inhibited by PP1 at concentrations (∼25 μM) similar to those that inhibit kinase activity in vivo (Figure S2A) (Wan et al., 2006). These experiments confirm that Ctf19-Mcm21, but not Ctf3, is a DDK substrate in vitro.

### DDK Phosphorylation Sites in the Ctf19 N-Terminal Region

We next sought to identify the Ctf19-Mcm21 residues targeted by DDK. A crystal structure of the Ctf19-Mcm21 dimer showed that N-terminal segments of both proteins are flexible (Schmitzberger and Harrison, 2012). We found that trypsin digestion of phosphorylated Ctf19-Mcm21 removed the phosphorylated residues under conditions that preferentially cleave the N-terminal tails from both proteins (Figure S2B), indicating that DDK phosphorylates residues in these unstructured regions.

Inspection of the N-terminal region of Ctf19 revealed three clusters of candidate phosphorylation sites (Figure 1C), and we found no candidates in the N-terminal region of Mcm21. DDK did not phosphorylate Ctf19-Mcm21 lacking the first 30 amino acids of Ctf19 (*ctf19*-Δ*30*), confirming that the DDK target site lies in this region. A Ctf19-Mcm21 complex in which Ctf19 was N-terminally tagged with MBP was also not phosphorylated in vitro, suggesting that residues very close to the N terminus of Ctf19 are important for phosphorylation by DDK. The serine and threonine residues near the Ctf19 N terminus are arranged in three near-repeat sequences, reminiscent of DDK target sites in Mcm4 and Rif1 (Hiraga et al., 2014, Sheu and Stillman, 2006). We mutated to alanine all candidate phosphate acceptors in the Ctf19 N-terminal region (*ctf19*-*9A*) or just a single candidate in each of the three near-repeat sequences (*ctf19*-*3A*-*1*) and tested for phosphate transfer to these complexes in vitro (Figures 1D and 1E). In all cases, phosphorylation was reduced to background levels. Mutation of just the first two candidate sites (T4, T5) to generate the *ctf19*-*2A* mutant (Figure 1D) did not abolish phosphorylation (Figure S2C). Finally, we also tested the effect of the Ctf3 complex on Ctf19 phosphorylation and found that it did not enhance phosphate transfer to Ctf19 in vitro (Figure S2D). We conclude that the Ctf19 N-terminal region is a specific substrate of DDK.

### Determinants of Ctf19 Phosphorylation In Vivo

**Figure 2:**
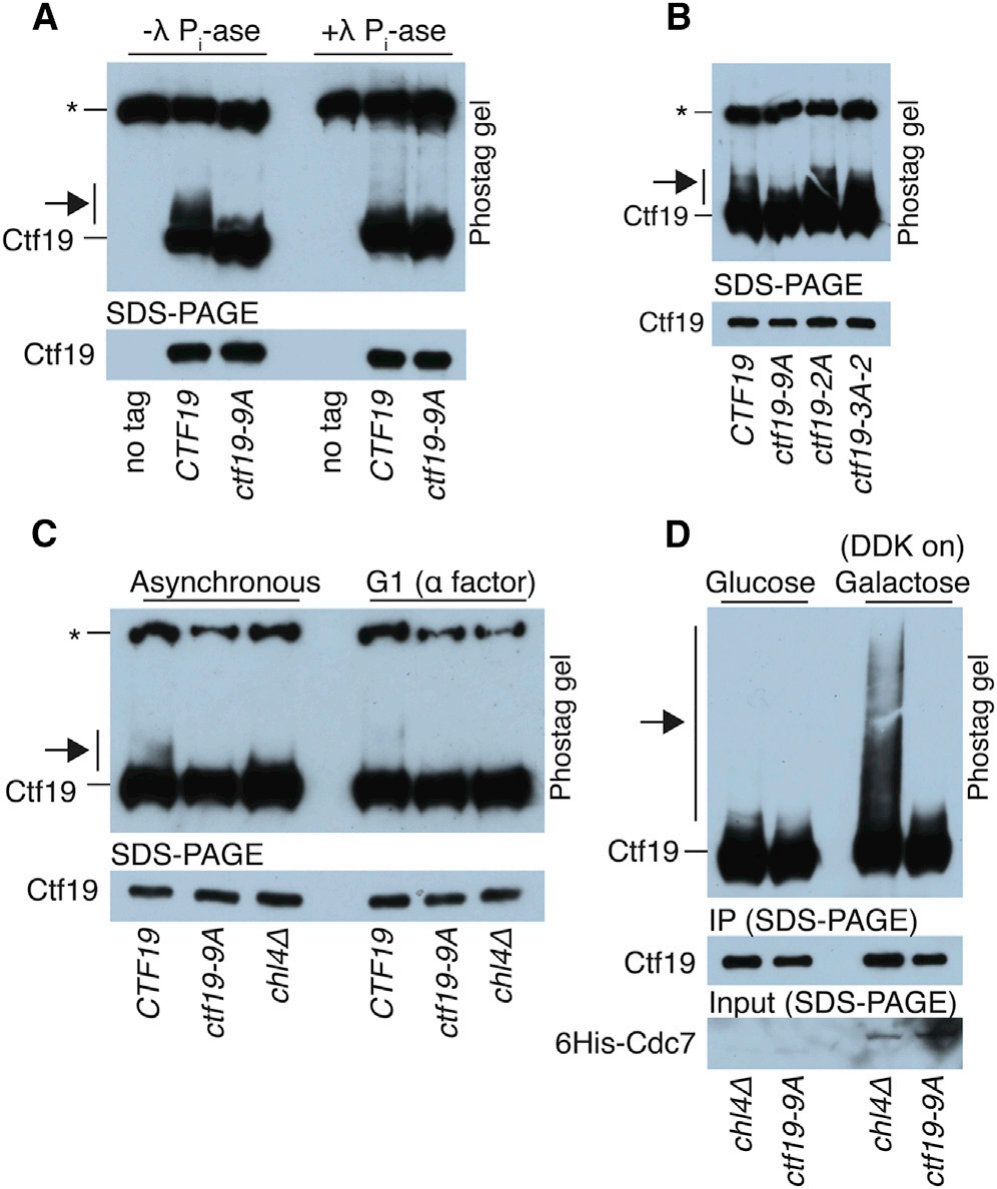
Observation of Ctf19 Phosphorylation In Vivo (A) Ctf19-3FLAG, purified from exponentially growing cells expressing either wild-type *CTF19* (SMH372) or *ctf19*-*9A* (SMH378) was resolved on a phostag gel and detected by western blot (arrow, phosphorylated Ctf19; ^∗^, non-specific band). (B) Ctf19 phosphorylation was assessed as in (A) for strains expressing *CTF19*, *ctf19*-*9A*, *ctf19*-*2A*, or *ctf19*-*3A*-*2* (SMH372, SMH378, SMH449, and SMH450, respectively). (C) Ctf19 phosphorylation assessed as in (A) for strains expressing *CTF19*, *ctf19*-*9A*, or *CTF19* in a *chl4*Δ background (SMH372, SMH378, and SMH401, respectively). Cells were untreated or arrested in G1 as indicated (arrows, phosphorylated Ctf19; ^∗^, non-specific band). (D) Strains bearing Ctf19-3FLAG and an inducible DDK expression cassette (Cdc7-6His, Dbf4 untagged) were not induced (left) or induced to express DDK (right) for 2 hr. Ctf19 phosphorylation was assessed in strains lacking *CHL4* (SMH429) or expressing *ctf19*-*9A* (SMH430).

**Figure S3:**
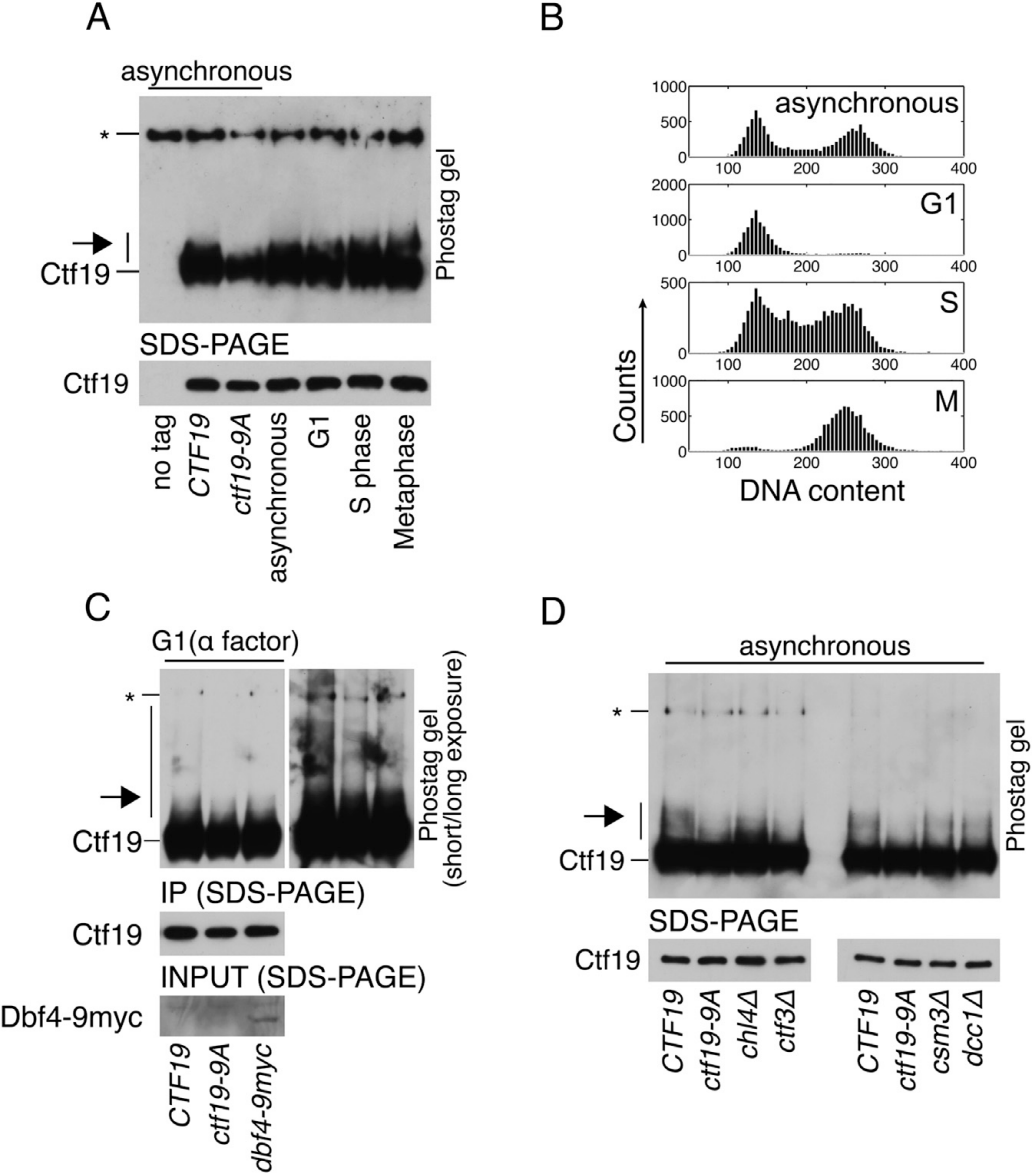
In Vivo Analysis of Ctf19 Phosphorylation, Related to Figure 2 (A) Ctf19 phosphorylation in arrested cultures (2h in arresting conditions; G1 – alpha factor; S phase – hydroxyurea; Metaphase – nocodazole and benomyl; arrows – phosphorylated Ctf19; ^∗^ – non-specific band). For lanes 1-3, the indicated strains were harvested during asynchronous growth (*CTF19* – SMH372, *ctf19-9A* – SMH378). For lanes 4-7, the *CTF19-3FLAG* strain used in lane 2 was harvested after the indicated arrests. (B) DNA content analysis for cultures used to produce samples 4-7 in panel B. (C) Ctf19 phosphorylation was assessed for strains expressing wild-type *CTF19* (SMH425), *ctf19-9A* (SMH426), or *CTF19* and *dbf4-9myc* (SMH427) and arrested in G1. Dbf4-9myc blot is shown for pre-immunoprecipitation samples. (D) Ctf19 phosphorylation was assessed for asynchronous cultures of the indicated strains (SMH372, SMH378, SMH401, SMH404, SMH402, and SMH403).

To test for phosphorylation in vivo, we purified Ctf19 from yeast and examined its mobility through a phostag-acrylamide matrix. We observed a slowly migrating species of wild-type Ctf19, which was susceptible to phosphatase treatment (Figure 2A, arrow). This Ctf19 mobility shift was evident in cells arrested in G1 (Figure 2C), S phase, and metaphase (Figures S3A and S3B). Ctf19 purified from *ctf19*-*9A* cells did not show this shift, indicating that the sites required for phosphorylation in vitro are also required for phosphorylation in vivo (Figure 2A). Mutating only the potential phosphorylation sites closest to the N terminus of Ctf19 (Figure 1D) did not eliminate the Ctf19 mobility shift (Figure 2B; Ctf19-2A; Ctf19-3A-2). Indeed, the Ctf19-2A protein with substitutions only at residues T4 and S5 was phosphorylated robustly in vivo as it was in vitro (Figures 2B and S2C).

To determine whether DDK localization is important for Ctf19 phosphorylation in vivo, we tested the effect of appending a C-terminal 9-myc tag to Dbf4, which perturbs DDK recruitment to the kinetochore in G1 (Natsume et al., 2013). Although the effect was subtle, we found decreased Ctf19 phosphorylation levels in this background (Figure S3C), supporting the idea that recruitment of DDK to kinetochores in G1 is important for robust Ctf19 phosphorylation. In the absence of Chl4, a Ctf19 complex protein required for DDK association with centromeres in G1 (Natsume et al., 2013), Ctf19 phosphorylation was only partly perturbed in asynchronous cultures but completely eliminated in G1-arrested cultures (Figures 2C and S3D). We conclude that the N-terminal region of Ctf19 is phosphorylated in vivo, and Chl4 determines the extent and timing of this phosphorylation.

If Chl4, which is not required for Ctf19 localization (Pot et al., 2003), determines Ctf19 phosphorylation levels by enabling DDK recruitment to kinetochores in G1, then overproduction of DDK should bypass this effect. To test this hypothesis, we overexpressed both DDK subunits from a galactose-inducible expression cassette and probed for Ctf19 phosphorylation (Figure 2D). Ctf19 phosphorylation was readily observable upon DDK overexpression in cells lacking Chl4 but not in the *ctf19*-*9A* strain. This result shows that Chl4 is dispensable for Ctf19 phosphorylation when DDK levels are not limiting.

In addition to the Ctf19 complex, robust centromere cohesion requires additional factors including Csm3 and Dcc1, members of the fork protection complex (FPC) and an alternative PCNA loading complex, respectively (Fernius and Marston, 2009, Mayer et al., 2004). Csm3 has a known role in DDK recruitment to the replisome in premeiotic S phase, suggesting a possible role in coordinating mitotic Ctf19 phosphorylation (Murakami and Keeney, 2014). We examined Ctf19 phosphorylation in *csm3*Δ and *dcc1*Δ strains and found it unperturbed in either background (Figure S3D), supporting the idea that these factors work in a pathway independent of cohesin loader recruitment (Fernius and Marston, 2009).

### The Ctf19 N-Terminal Region Is Not Required to Recruit DDK or Replication Factors

**Figure 3:**
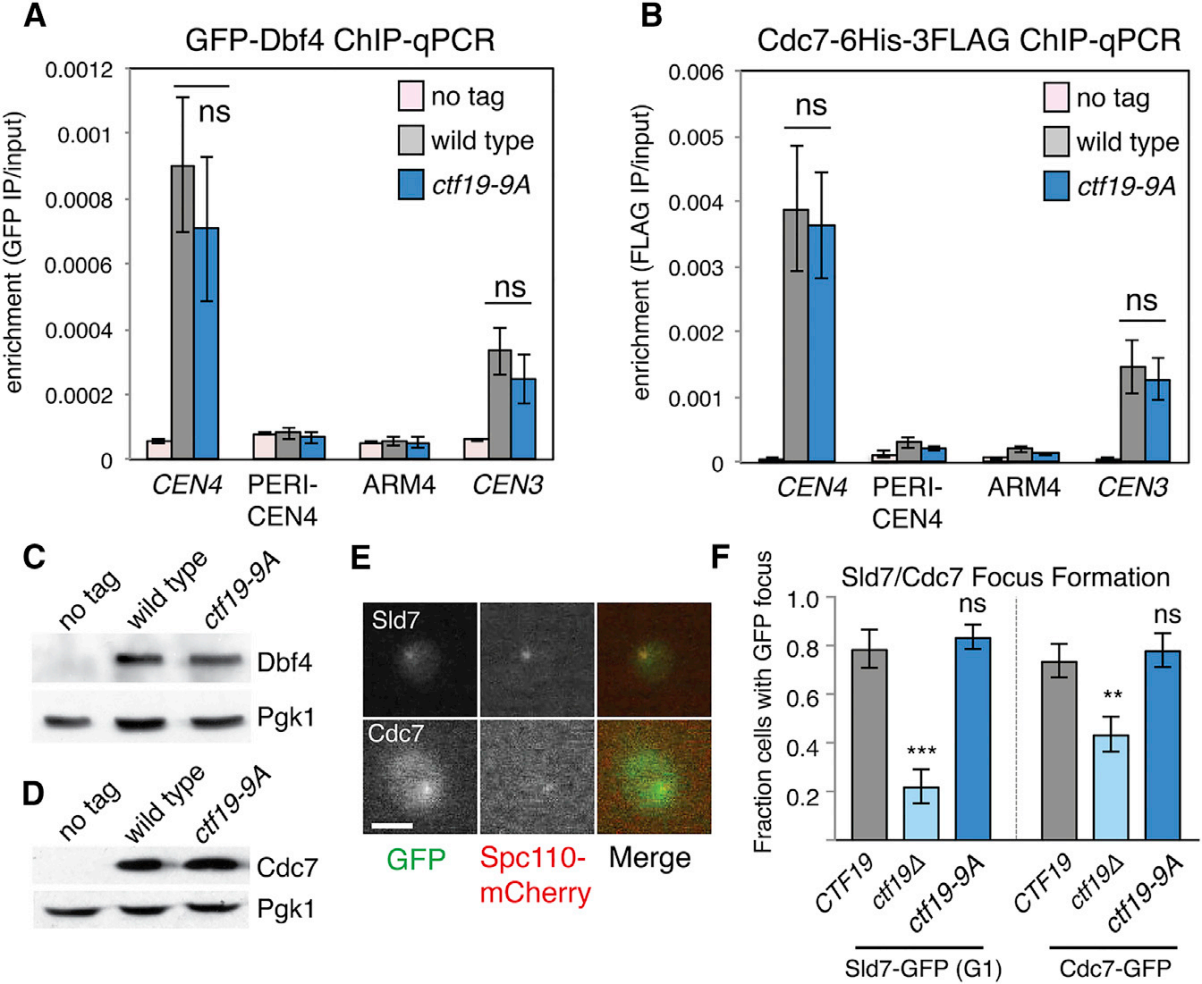
Ctf19 Phosphorylation Is Not Required for DDK Localization or Early Origin Activation (A and B) DDK localization is not perturbed by *ctf19*-*9A.* Wild-type and *ctf19-9A* cells carrying *GFP*-*DBF4* (AM21871 and AM21872, respectively (A), or *CDC7*-*6His*-*3FLAG* (AM21110 and AM21234, respectively (B), together with an untagged control strain (AM1176) were arrested in G1 with alpha factor for 3 hr before harvesting for ChIP-qPCR (average of three biological repeats, error bars show SE; ns, not significant, Student’s t test). (C and D) Dbf4 or Cdc7 protein levels are unaltered in *ctf19*-*9A* strains. Representative anti-GFP and anti-HA immunoblots are shown (Pgk1, loading control). (E and F) Live-cell imaging to assess Sld7 and Cdc7 localization. Strains expressing Spc110-mCherry to mark spindle pole bodies and either Sld7-GFP or Cdc7-GFP were examined for the presence of GFP foci in G1 (SMH477, SMH478, and SMH480 for Sld7-GFP; SMH473, SMH474, and SMH476 for Cdc7-GFP). Sld7-GFP-expressing strains were arrested in G1 with alpha factor, and Cdc7-GFP-expressing strains were observed during unperturbed growth. (E) Images of wild-type *CTF19* cells in G1 are shown for Sld7-GFP (top) and Cdc7-GFP (bottom; scale bar, 3 μm). (F) Fraction of cells with foci in the indicated strains (error bars, +/−SD for three experiments; ^∗∗^p < 0.01; ^∗∗∗^p < 0.001; ns, not significant; Student’s t test versus the *CTF19* strain for the indicated GFP fusion protein, two tails, unequal variance). See also Figure S4.

Ctf19 is required for centromeric DDK recruitment and the subsequent early replication of centromeric DNA (Natsume et al., 2013), raising the possibility that the Ctf19 N-terminal region is not merely a substrate, but also a recruiter of DDK. To test whether the *ctf19*-*9A* allele interferes with proper DDK recruitment, we assessed DDK localization in cells arrested in G1 with alpha factor by performing chromatin immunoprecipitation (ChIP)-qPCR for the Dbf4 and Cdc7 subunits of the kinase complex (3GFP-Dbf4 and Cdc7-6His-3FLAG, respectively; Figures 3A–3D). We found that DDK localization to centromeres was independent of Ctf19 N-terminal region phosphorylation, a finding confirmed by imaging Cdc7-GFP in live cells (Figures 3E and 3F). Furthermore, Sld7-GFP, a replication origin factor that depends on Ctf19 for its centromeric localization in G1 (Natsume et al., 2013), was also recruited normally to centromeres in the *ctf19*-*9A* background (Figures 3E and 3F). We conclude that Ctf19 phosphorylation is dispensable for DDK recruitment to centromeres and early origin activation.

### Ctf19 Phosphorylation Is Required to Recruit Scc2 to Centromeres

**Figure 4:**
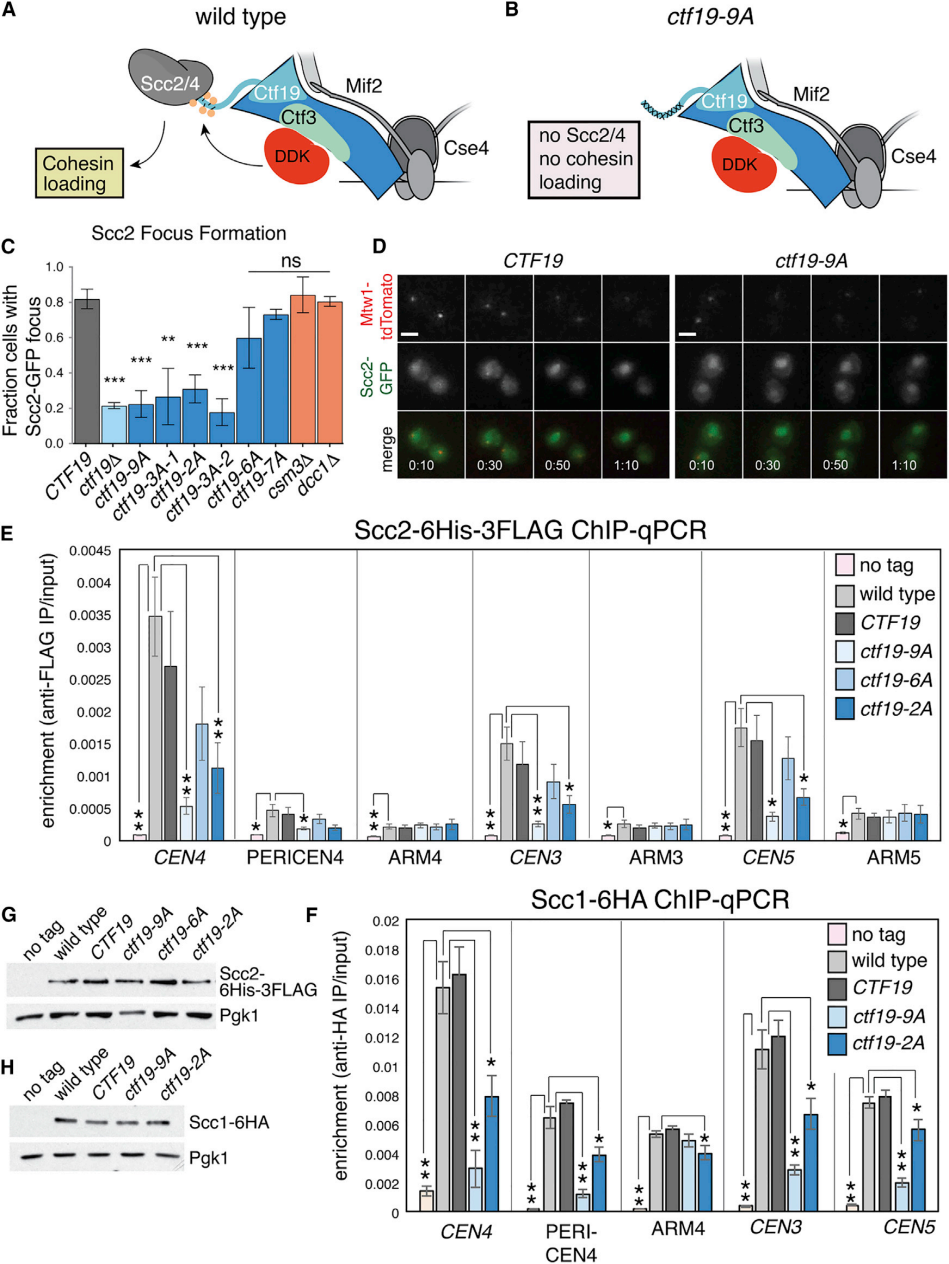
Ctf19 Phosphorylation Is Required for Scc2 Localization at Centromeres (A and B) Model for the mechanism of Scc2/4 (gray) recruitment by the Ctf19 complex (blue) and DDK (red). Ctf3 is shown in green, and phosphate modifications are depicted as yellow circles. (A) Ctf3 recruits DDK, which phosphorylates Ctf19 to provide a docking site for Scc4. (B) Cells lacking the Ctf19 phosphorylation sites lack the Scc4 docking site, despite normal DDK recruitment. (C and D) Ctf19 phosphorylation is required for efficient localization of Scc2 to centromeres. Strains of the indicated genotypes expressing Scc2-GFP and Mtw1-tdTomato (SMH353, SMH361, SMH354, SMH355, SMH451, SMH452, SMH459, SMH460, SMH416, and SMH417 listed in the order shown) were imaged during unperturbed vegetative growth and scored for the presence of centromeric Scc2-GFP foci. Values are the averages of at least three independent experiments. At least 25 cells were scored per strain per experiment (error bars, +/−SD for the independent experiments; ^∗∗∗^p < 0.001; ^∗∗^p < 0.01; ns, not significant; Student’s t test versus CTF19 strain, two tails, unequal variance). (D) Representative images from experiments quantified in (C) (scale bar, 3 μm). Time after the beginning of image acquisition is shown. (E and F) Scc2 and Scc1 localization require Ctf19 phosphorylation. Cells were arrested in metaphase following treatment with nocodazole and benomyl for 2.5 hr (AM1176, untagged control strain). Values are means from four independent experiments (error bars, SE; ^∗^p < 0.05; ^∗∗^p < 0.01; Student’s t test for indicated comparisons, unpaired, two-tails). (E) ChIP-qPCR for Scc2 performed using wild-type (AM6006), *CTF19* (AM20613), *ctf19*-*9A* (AM20619), *ctf19*-*6A* (AM20622), and *ctf19*-*2A* (AM21908) cells, carrying SCC2-*6His*-*3FLAG*. (F) ChIP-qPCR for Scc1 performed using wild-type (AM1145), *CTF19* (AM20629), *ctf19*-*9A* (AM23456), and *ctf19*-*2A* (AM21958) cells, carrying *SCC1*-*6HA*. (G and H) Immunoblots showing levels of Scc2-6His-3FLAG, Scc1-6HA, and Pgk1 (loading control) from a representative experiment.

Kinetochore-localized DDK and the Ctf19 complex enable cohesin loading at centromeres by recruiting Scc2/4. Our findings that the Ctf19 N-terminal region is a substrate of DDK and the Ctf3 complex is a DDK binding partner suggested that phosphorylation of Ctf19 might create a docking site for the Scc2/4 cohesin loader at the kinetochore (Figures 4A and 4B). To test this idea, we imaged cells expressing Scc2-GFP as they progressed through an unperturbed cell cycle (Figures 4C and 4D). Under these conditions, Scc2-GFP forms a kinetochore-associated focus as wild-type cells enter S phase (Fernius et al., 2013, Natsume et al., 2013). We found that Scc2-GFP did not make foci at kinetochores (identified by Mtw1-tdTomato in our experiments) in the *ctf19*-*9A* background, while kinetochore localization was readily observable in the wild-type background. This effect was specific for Ctf19 mutants, as Scc2-GFP localization was unperturbed in *csm3*Δ and *dcc1*Δ cells (Figure 4C).

We also used ChIP-qPCR to assess the localization of Scc2 (Figures 4E and 4G). Consistent with the imaging, we found that Scc2 occupancy at three test centromeres was reduced in the *ctf19*-*9A* strain to the levels seen at arm sites. Reduced Scc2 recruitment to centromeres is expected to result in decreased cohesin levels throughout the pericentromere. Indeed, ChIP-qPCR from the *ctf19*-*9A* mutant showed reduced levels of Scc1 at centromeric and pericentromeric sites but not at chromosomal arm sites (Figures 4F and 4H). Therefore, Ctf19 phosphorylation is required for Scc2 recruitment to centromeres and for cohesin enrichment in the pericentromere.

### A Subset of Phosphorylated Ctf19 Residues Recruits Scc2 to Centromeres

Elevated conservation of the two phosphorylation sites closest to the N terminus of Ctf19 raised the possibility that these sites might be especially important for Scc2/4 recruitment to the kinetochore (Figure 1C), even though they are not the only sites phosphorylated in vivo (*ctf19*-2A; Figures 2B and S2C). To test this hypothesis, we examined Scc2-GFP localization in cells in which only the first three (T4A, S5A, T7A; *ctf19*-*3A*-*2*) or two (T4A, S5A; *ctf19*-*2A*) Ctf19 phosphate acceptor residues had been mutated. Like *ctf19*-*9A* cells, strains bearing either the *ctf19*-*3A*-*2* or the *ctf19*-*2A* allele did not efficiently localize Scc2 to centromeres as determined by fluorescence imaging (Scc2-GFP; Figure 4C) and by ChIP-qPCR (Figure 4E). Accordingly, cohesin recruitment to centromeres was diminished in the *ctf19*-*2A* strain, though less severely than in the *ctf19*-*9A* strain (Figure 4F). To determine whether phosphorylation of the residues closest to the Ctf19 N terminus is sufficient for Scc2/4 recruitment to kinetochores, we examined the effects of Ctf19 alleles in which all other Ctf19 phosphate acceptors had been mutated to alanine. The *ctf19*-*6A* strain, which is identical to *ctf19*-*9A* except that Ctf19 residues T4, S5, and T7 are unmutated, had only a mild defect in centromeric Scc2 occupancy (Figure 4C, E). Together, these findings show that phosphorylation of Ctf19 residues T4, S5, and T7 is both necessary and sufficient to drive Scc2/4 localization to kinetochores.

### Phosphorylation of Ctf19 Is Required for Robust Centromere Cohesion

**Figure 5:**
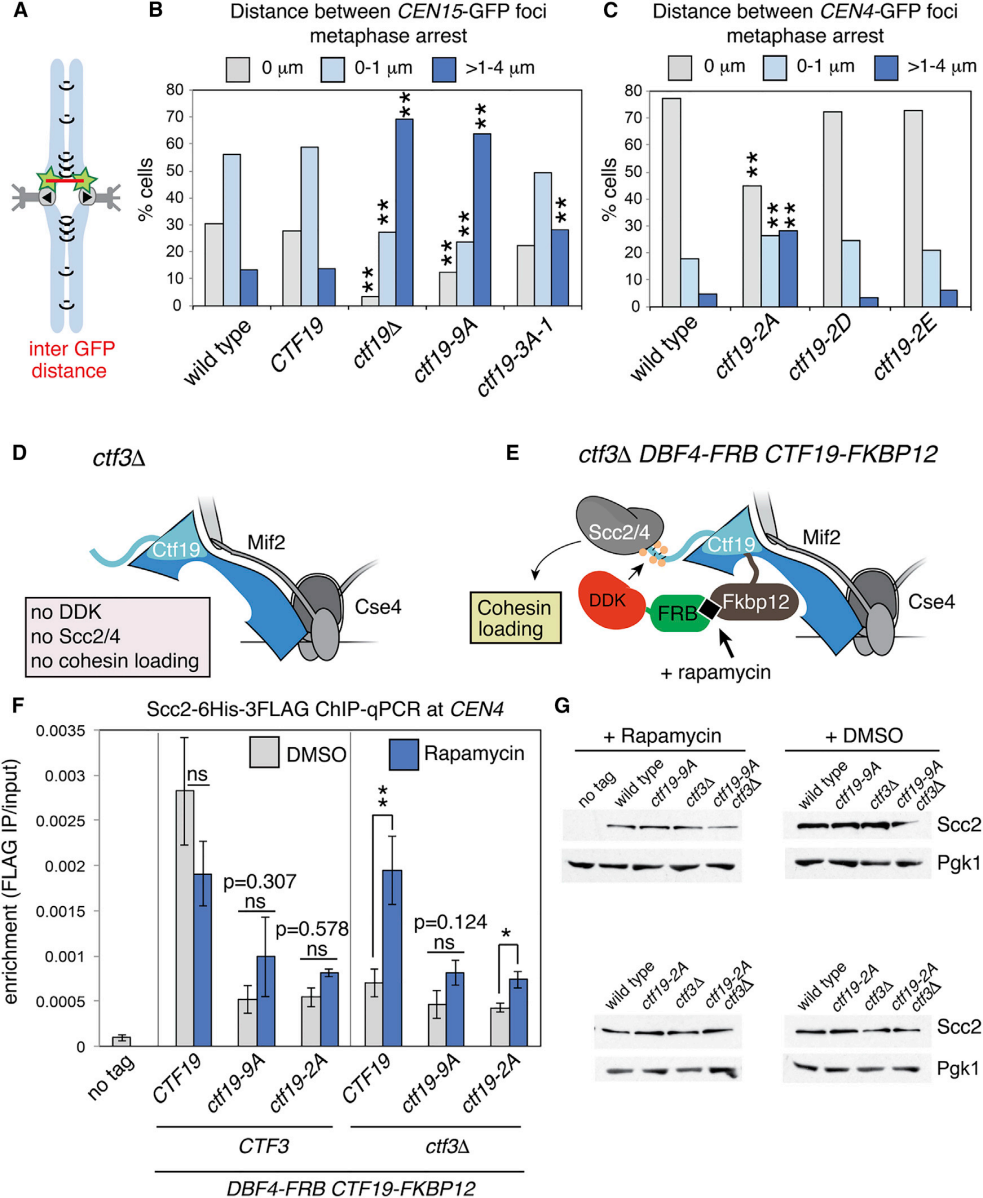
Ctf19 Phosphorylation Is Required for Centromeric Cohesion Establishment (A) Schematic depicting sister centromere separation at metaphase. Distance between centromere-associated GFP foci (green stars) was measured (red line). (B and C) Sister centromeres are further apart in metaphase in *ctf19*-*9A* cells compared to wild-type cells. (B) Strains bearing a *lacO* array at *CEN15* (1.8 kb to the left of *CEN15*) and expressing LacI-GFP were imaged after arrest in metaphase by depletion of Cdc20 (*pMET*-*HA3*-*CDC20*) for 2h. Average centromere separation was determined for wild-type (SMH159), *CTF19* (SMH412), *ctf19*Δ (SMH395), *ctf19*-*9A* (SMH396), and *ctf19*-*3A*-*1* (SMH397) strains. Values were binned into categories (0 μm for no separation, 0–1 μm, and >1 μm–4 μm) and expressed as a percentage of all cells scored across three experiments. The fraction of cells in each bin was compared to the wild-type using Fishers exact test (^∗∗^p < 0.0001). (C) Phospho-null but not phospho-mimetic mutations in phosphorylation sites closest to the Ctf19 N terminus cause a centromere cohesion defect. Wild-type (AM4643), *ctf19*-*2A* (AM22083), *ctf19*-*2D* (AM22481), and *ctf19*-*2E* (AM22484) strains, all carrying *+2.4 CEN4*-*GFP* (2.4 kb to the right of *CEN4*) were arrested in G1 before release into medium containing methionine to deplete *CDC20* for 2 hr before imaging and analyzed as in (B). (D and E) Schematic showing the cohesin loading defect in *ctf3*Δ cells (D) and rescue of this defect by artificial tethering of Dbf4-FRB to the kinetochore (E). (F) Scc2 localization was assessed by ChIP-qPCR in the presence or absence of DDK tethering, *CTF3*, and Ctf19 phosphorylation sites. Wild-type (AM21613), *ctf19*-*9A* (AM21681), *ctf19*-*2A* (AM22360), *ctf3*Δ (AM21614), *ctf19*-*9A ctf3*Δ (AM21617), *ctf19*-*2A ctf3*Δ (AM22672), and control (AM21486) strains, all expressing *DBF4*-*FRB* and *CTF19*-*FKBP12*, were arrested in G1 for 3 hr before release into medium containing nocodazole, benomyl, and either DMSO (no DDK tethering) or rapamycin (DDK tethering; 1 μM) for 2.5 hr. Mean values of at least four independent experiments are shown. Error bars indicate SE (^∗^p < 0.05; ^∗∗^p < 0.01; Student’s t test, unpaired, two-tails; ns, not significant). (G) Immunoblots showing relative levels of Scc2-6His-3FLAG and Pgk1 (loading control) in a representative experiment.

To determine whether failure to target Scc2 to centromeres upon mutation of the phosphorylation sites in Ctf19 results in defective centromere cohesion, we examined centromere separation in cells arrested in metaphase by Cdc20 depletion (Figures 5A–5C). In cells carrying the *ctf19*-*9A* allele, the distance between centromeric GFP foci was comparable to that in the *ctf19*Δ strain (Figure 5B). Alanine substitutions at a single phosphate acceptor site in each of the three N-terminal repeat-like sequences in Ctf19 (S5A, T8A, S14A; *ctf19*-*3A*-*1*) resulted in a partial centromere cohesion defect (Figure 5B).

Because phosphorylated sites near the N terminus of Ctf19 appear most critical for Scc2/4 recruitment, we examined the effect on centromere cohesion of mutating T4 and S5 to phospho-null (alanine; *ctf19*-*2A*) or phosphomimetic (aspartate; *ctf19*-*2D* and glutamate; *ctf19*-*2E*) residues (Figure 1D). While *ctf19*-*2A* resulted in a significant centromere cohesion defect, the phospho-mimetic *ctf19*-*2D* and *ctf19*-*2E* mutants did not (Figure 5C), consistent with the idea that it is phosphorylation of Ctf19 residues near its N terminus that enables centromere cohesion.

### Scc2 Recruitment by Artificially Tethered DDK Depends on Ctf19 N-Terminal Phosphorylation Sites

**Figure S4:**
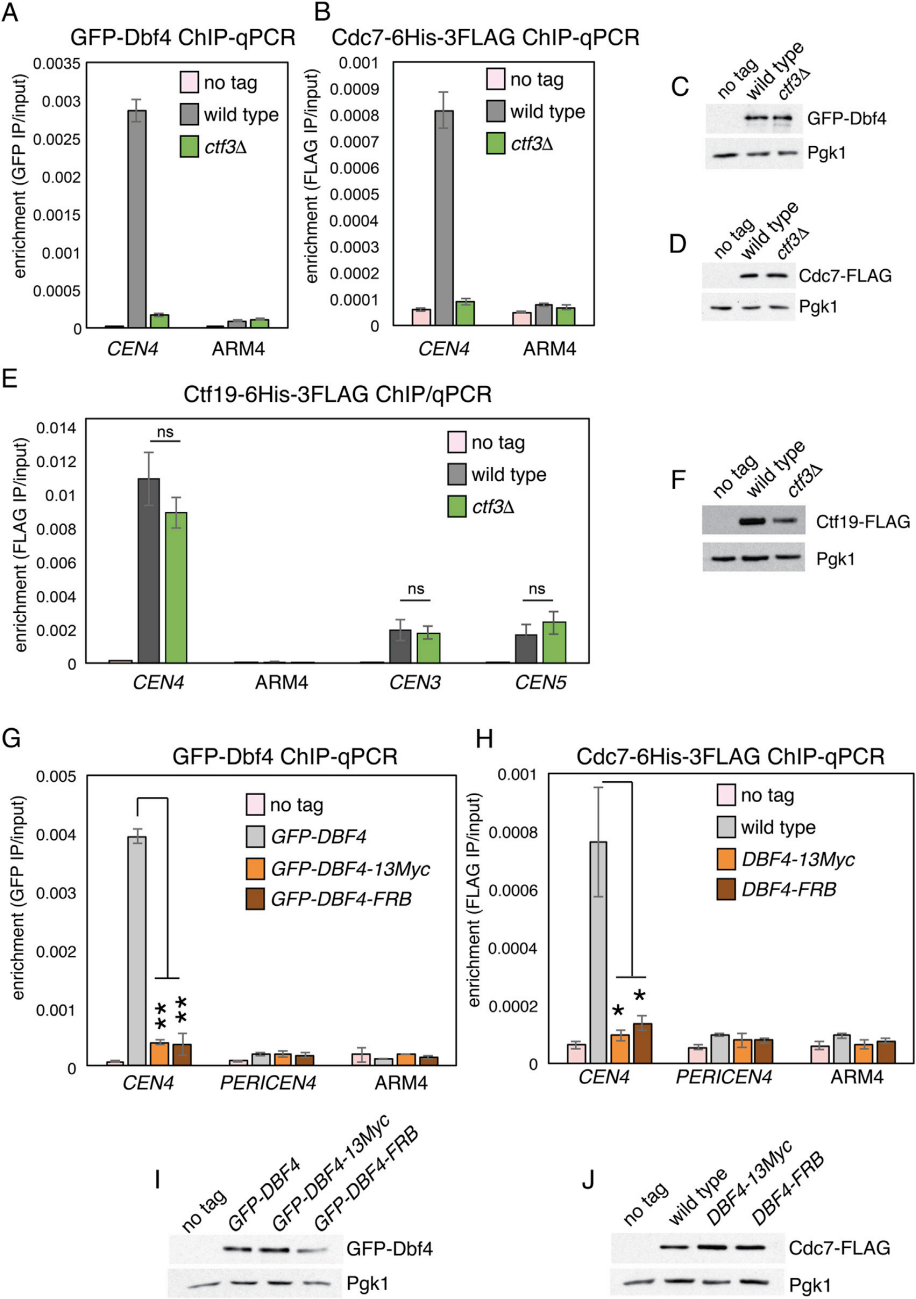
DDK Recruitment to Kinetochores Is Abolished in Cells Lacking *CTF3* or with C-Terminally Tagged Dbf4, Related to Figure 3 (A and B) DDK localization was assessed by ChIP-qPCR for wild-type and *ctf3Δ* strains. Wild-type and *ctf3Δ* cells carrying *GFP-DBF4* (AM21871 and AM23275, respectively; panel A) or *CDC7-6His-3FLAG* (AM21110 and AM23268, respectively; panel B), together with an untagged control strain (AM1176) were arrested in G1 for 3h before harvesting for ChIP-qPCR. Means of three independent experiments are shown (error bars – standard error). (C and D) Immunoblots showing levels of GFP-Dbf4, Cdc7-FLAG and Pgk1 (loading control) in the samples from a representative experiment. (E) Ctf19 localization was assessed by ChIP-qPCR for wild-type and *ctf3Δ* strains. Wild-type and *ctf3Δ* strains carrying *CTF19-6His-3FLAG* were arrested in metaphase by treatment with nocodazole and benomyl and ChIP-qPCR experiments were performed as in Figure 5F. (F) Immunoblot showing levels of Ctf19-6His-3FLAG and Pgk1 (loading control) for the indicated strains. (G and H) DDK localization was assessed by ChIP-qPCR for strains expressing C-terminally tagged Dbf4. Wild-type cells and those in which Dbf4 bears a C-terminal 13Myc or FRB tag and carrying *GFP-DBF4* (AM21871, AM22226, and AM22230, respectively; panel E) or *CDC7-FLAG* (AM21110, AM22538, and AM22540, respectively; panel F), along with an untagged control strain (AM1176), were arrested in G1 for 3h before harvesting for ChIP-qPCR. The means of three independent experiments are shown (error bars – standard error; ^∗^ – p < 0.05; ^∗∗^ – p < 0.01; Student’s t test, paired, two-tails). (I and J) Immunoblots showing relative levels of GFP-Dbf4, Cdc7-FLAG and Pgk1 (loading control) in the samples from a representative experiment.

Our in vitro binding studies (Figure S1) indicated that, in addition to interacting with its substrate Ctf19, DDK also associates with Ctf3, which is not a substrate. ChIP-qPCR showed that the amounts of both Cdc7 and Dbf4 are strongly reduced at centromeres in G1-arrested *ctf3*Δ cells (Figures S4A–S4D). To test the hypothesis that the critical role of Ctf3 in centromeric cohesin loading is to enable Ctf19 phosphorylation by recruiting DDK to centromeres, we sought to artificially recruit DDK to centromeres in cells lacking Ctf3. We tagged Ctf19 with FKBP12 and Dbf4 with FRB, so that upon rapamycin addition, Dbf4-FRB would be tethered to Ctf19-FKBP12 at the kinetochore. Because kinetochore localization of Ctf19 does not depend on Ctf3 (Figures S4E and S4F) (Pot et al., 2003), forced Dbf4-Ctf19 association should bypass the normal requirement for Ctf3 in centromeric DDK recruitment. Consistent with a previous study (Natsume et al., 2013), tagging Dbf4 at its C terminus with either 13Myc or FRB diminished its association with centromeres in G1 cells (Figures S4G–S4J). However, centromeric Scc2 occupancy in metaphase cells producing Dbf4-FRB was comparable to that of wild-type cells, even in the absence of rapamycin (Figure 5F; *CTF19*, DMSO condition; compare with Figure 4E, wild-type). It is therefore likely that Dbf4 tagged at its C terminus with FRB retains the ability to access its Ctf19 substrate, at least during metaphase. Centromeric Scc2 localization in Dbf4-FRB cells in the absence of rapamycin nonetheless depended on Ctf3, while artificial tethering of Dbf4-FRB to Ctf19-FKBP12 by addition of rapamycin bypassed the Ctf3 requirement (Figure 5F). Thus, artificial tethering of Dbf4 to Ctf19 can substitute for Ctf3 in recruiting Scc2 to centromeres. Furthermore, mutation of phosphorylation sites near the Ctf19 N terminus completely (*ctf19*-9A) or partially (*ctf19*-2A) prevented tethered Dbf4 from restoring Scc2 association with centromeres in *ctf3*Δ cells (Figure 5F). We conclude that Ctf3 recruits DDK, which in turn phosphorylates Ctf19, thereby enabling Scc2 association with centromeres and cohesin loading.

### Phosphorylated Ctf19 Binds Scc4

**Figure 6:**
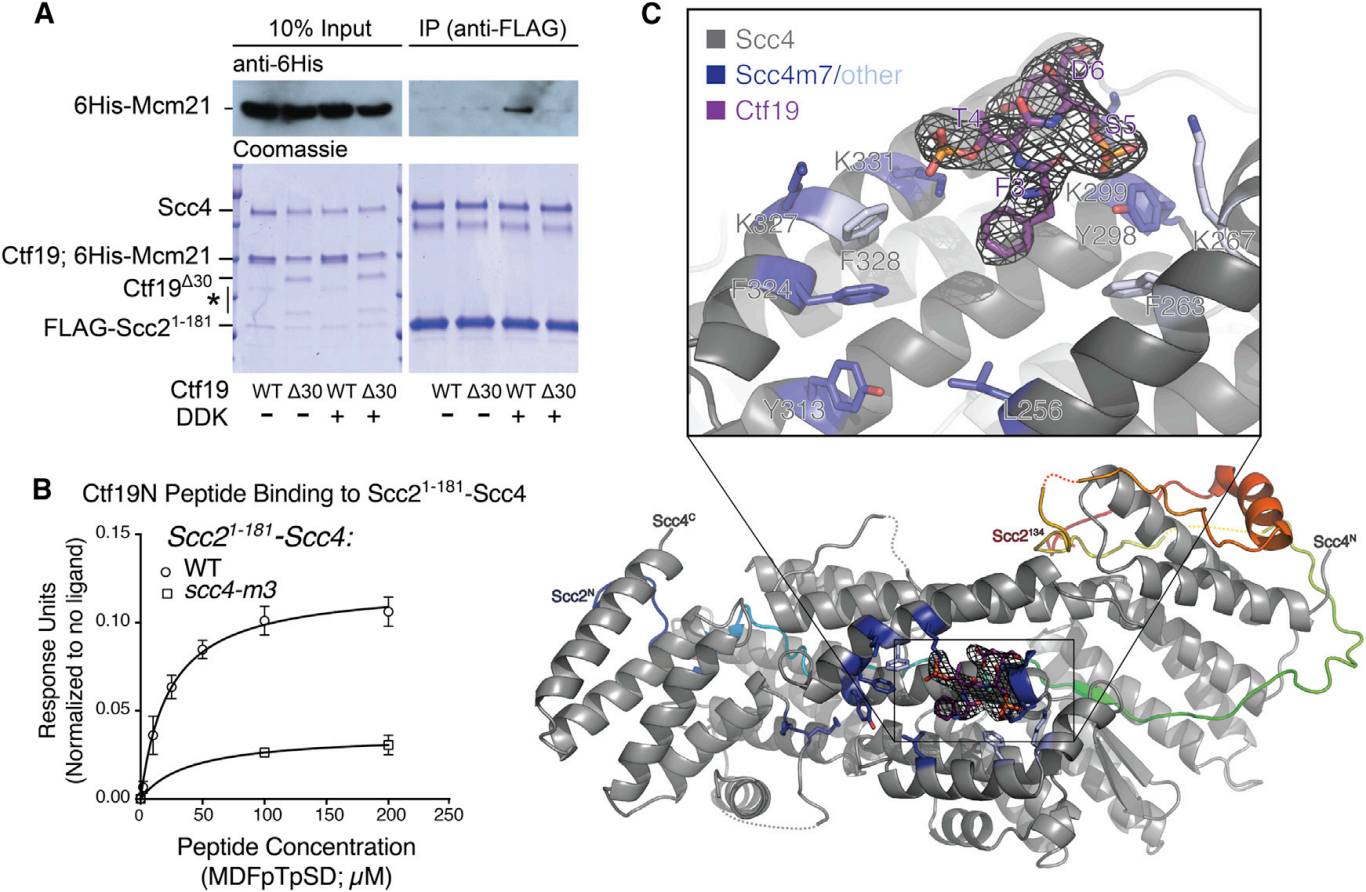
Scc4 Recognizes Phosphorylated Ctf19 (A) Reconstitution of DDK-dependent Scc4-Ctf19 interaction in vitro. Recombinant Ctf19-6His-Mcm21 complexes were phosphorylated with purified DDK and then used for pulldowns with recombinant FLAG-Scc2^1–181^-Scc4. Proteins were immunopurified on anti-FLAG beads, resolved by SDS-PAGE, and detected by immunoblot against an 6His-Mcm21 (top) or by Coomassie stain (total protein, bottom; ∗, degradation product). (B) Synthetic Ctf19^1–6^ peptide (MDFpTpSD) was tested for binding to Scc2^1–181^-Scc4^WT^ or Scc2^1–181^-Scc4^m3^. Mean average scaled response units are shown for three independent experiments for each data point. (C) Structure of phosphorylated Ctf19^1–6^ bound to Scc2^1–181^-Scc4. An overview of the complex (bottom) is shown as a cartoon with Scc4 in gray and Scc2^1-181^ in rainbow (violet, N terminus; red, C terminus). The Ctf19-Scc4 interaction is shown in detail above. Individual residues mutated in the *scc4*-*m7* allele are in purple. Other residues contributing to the interaction are in light blue. Ctf19 is purple with non-carbon atoms colored by element (red, oxygen; blue, nitrogen; yellow, phosphorous). Omit map density contoured to 0.8 sigma is shown for the Ctf19 peptide. See also Figure S5 and Tables S2 and S3.

Scc4 is the localization module of the cohesin loading complex, but an interaction with kinetochore proteins has not yet been observed. To test the idea that phosphorylated Ctf19 is the centromeric receptor for Scc2/4, we attempted to reconstitute Scc2/4 targeting in vitro. Using a pulldown assay with recombinant Scc2^1–181^-Scc4 as bait and Ctf19-Mcm21 as prey (Figure 6A), we found that Ctf19-Mcm21, when treated with DDK and ATP, associated with Scc2^1–181^-Scc4, and this interaction required the N-terminal region of Ctf19. Therefore, Ctf19, when phosphorylated by DDK, is a direct binding partner of Scc2/4.

**Figure S5:**
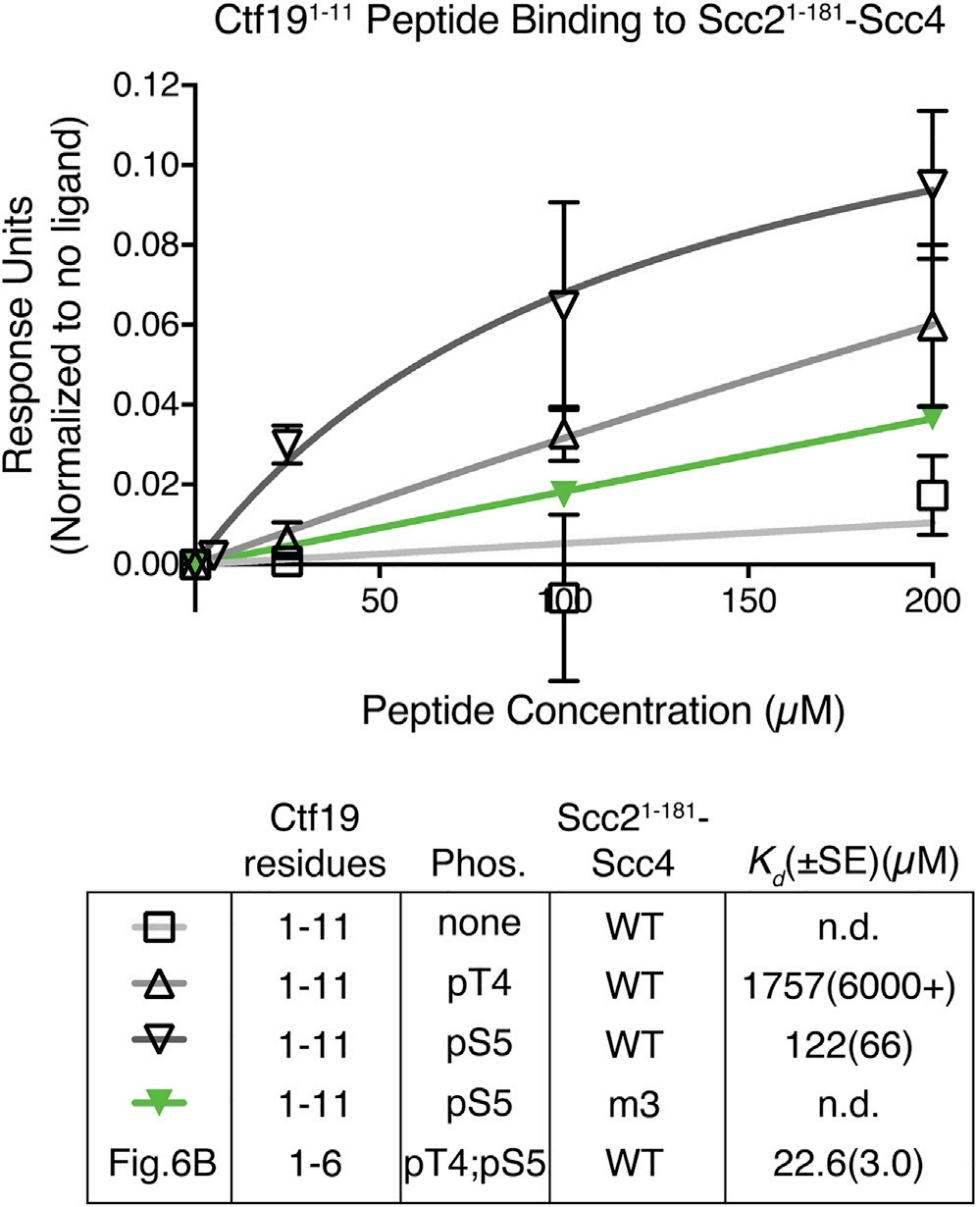
Synthetic Ctf19^1–11^ binds Scc2^1–181^-Scc4, and Binding Requires Phosphorylation of Ctf19 Residue T4 or S5, Related to Figure 6 Experiments were carried out as in Figure 6B (error bars – +/−SD for three independent experiments, obscured by data points where not visible). m3 refers to immobilized Scc2^1-181^-Scc4^m3^. Ctf19 peptides and Scc2^1-181^-Scc4 complexes used are listed in the table along with measured dissociation constants (*K*_*d*_; SE – standard error for best-fit binding curve; single site, specific binding; Phos. – phosphorylated residues).

To determine whether the N-terminal region of Ctf19 is sufficient for Scc4 binding, we tested whether synthetic Ctf19 peptides interact with recombinant Scc2^1–181^-Scc4. We found that Ctf19^1–6^ phosphorylated at amino acid residues T4 and S5 bound Scc2^1–181^-Scc4 in vitro, while an unphosphorylated peptide did not (Figures 6B and S5). The weak in vitro affinity (dissociation constant [Kd] ∼23 μM) may reflect the absence of stabilizing factors present in vivo. To determine whether this interaction depended on the conserved surface of Scc4 required for kinetochore recruitment of Scc2 (Hinshaw et al., 2015), we performed identical experiments with a mutant version of the Scc2^1–181^-Scc4 complex (Scc2^1–181^-Scc4^m3^; Figure 6B). This Scc4 mutant perturbs centromeric cohesin loading in vivo, and we found it deficient in its interaction with the phosphorylated Ctf19 peptide in vitro. We also tested longer Ctf19 peptides bearing at most one phosphate group for binding with Scc2^1–181^-Scc4 (Figure S5). We found that unmodified Ctf19^1–11^ did not interact with Scc2^1–181^-Scc4. This observation is consistent both with the dependence of centromeric cohesin loading on DDK and with our inability to detect an interaction between Ctf19-Mcm21 and Scc2/4 using unmodified recombinant protein complexes expressed in bacteria (Figure 6A). Single phosphorylation at either T4 or S5 of Ctf19 resulted in detectable binding, with phosphorylation at S5 providing a higher apparent affinity (Figure S5). We conclude that the N-terminal segment of Ctf19, once it has been phosphorylated by DDK, specifically interacts with the conserved surface patch of Scc4. Furthermore, phosphorylated Ctf19 residues T4 and S5 each contribute to this interaction, at least in vitro. This finding is consistent with our observation that Ctf19 residues T4 and S5 (along with T7) are the necessary and sufficient DDK targets for centromeric Scc2/4 localization (Figures 4C and 4E). Our binding studies also explain the observation that mutation of Ctf19 serine 5 to alanine without mutation of threonine 4 leads to a partial but not complete cohesion defect (*ctf19*-*3A*-*1*, Figure 5B).

### Structural Basis of Ctf19 Recognition by Scc4

To observe the interaction between phosphorylated Ctf19 and Scc4 directly, we determined the crystal structure of a phosphorylated Ctf19 N-terminal peptide (MDFpTpSD) bound to Scc2^1–181^-Scc4 (Figure 6C). Inclusion of the Ctf19 peptide yielded crystals in a different space group than the one we had reported previously for unliganded Scc2^1–181^-Scc4 (PDB: 4XDN), with protomers now packing so that the Scc4 conserved patch was far enough from crystallographic symmetry mates to allow occupancy by a small peptide. Iterative density modification with non-crystallographic symmetry averaging revealed extra density near the Scc4 conserved patch, allowing us to model key Ctf19 residues, including the conserved F3 and the phosphorylated T4 and S5 residues.

This crystal structure explains both the results of our binding studies using synthetic Ctf19 peptides and the behavior of the *ctf19*-*2A* and *ctf19*-*6A* alleles in vivo. The phosphorylated Ctf19 residues T4 and S5 make electrostatic contacts with lysine side chains protruding from the Scc4 surface required for centromere cohesion (Scc4 K331 and K267). Coordination of these phosphates traps Ctf19 F3 between two aromatic side chains deep within the Scc4 conserved pocket (Scc4 F328 and F263). The structure also explains why the *scc4*-*m3*, *scc4*-*m35*, and *scc4*-*m7* alleles abrogate Scc2 targeting to centromeres (Hinshaw et al., 2015). The conserved residues F324 and K331 are mutated in all three alleles. In addition to direct coordination of the phosphate at Ctf19 T4 by Scc4 K331, residue F324 stabilizes the position of Scc4 F328 and therefore aids in recognition of Ctf19 F3. Scc4 residues Y298 and K299 were mutated in the *scc4*-*m7* allele. Y298 helps coordinate the phosphate at Ctf19 S5, and K299 makes contact with the Ctf19 peptide backbone, explaining the contribution of these residues to the *scc4*-*m7* allele.

### Conservation of Scc4-Mediated Scc2 Targeting in Vertebrates

**Figure 7:**
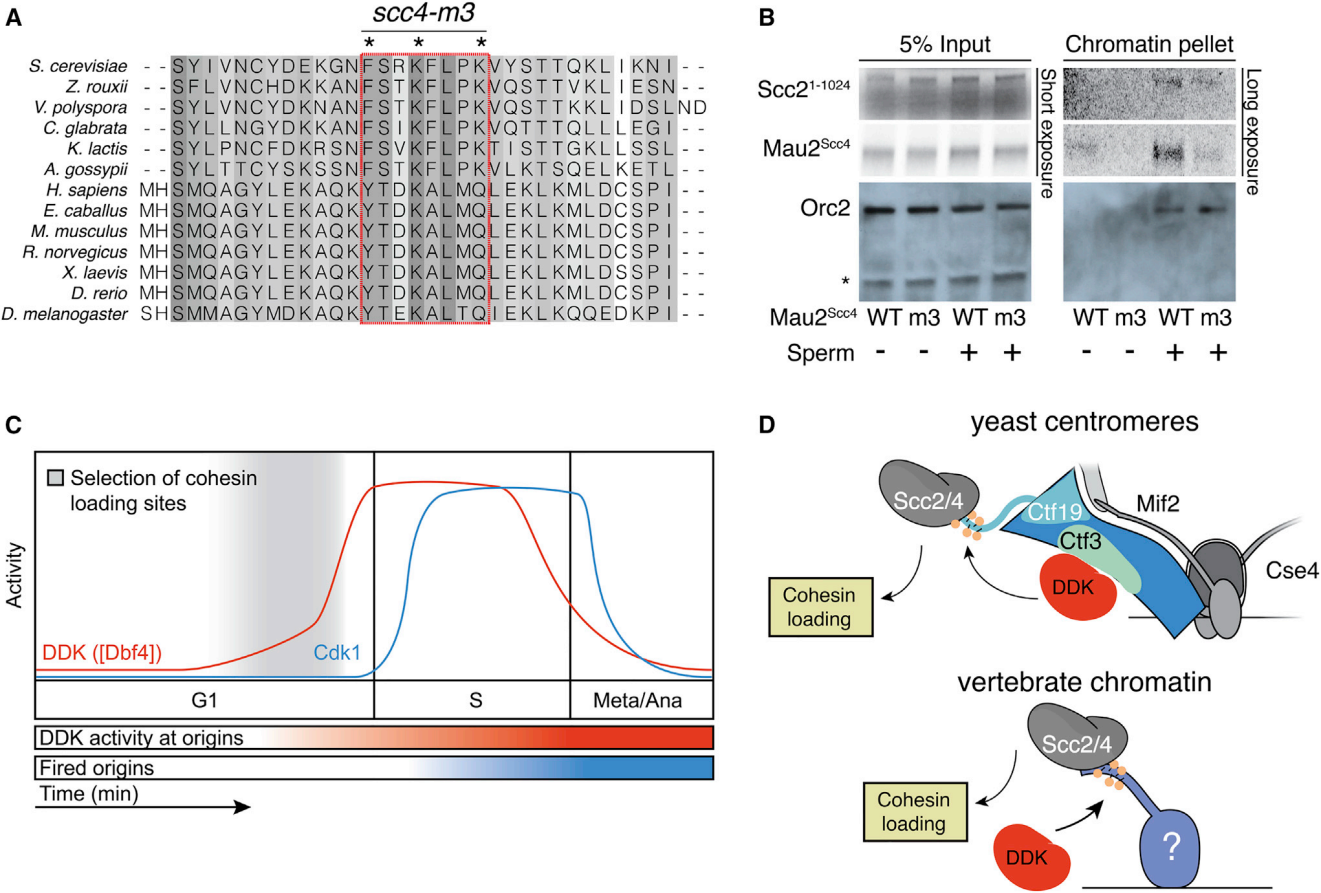
Scc4-Dependent Scc2/4 Localization in Vertebrates (A) Multiple sequence alignment for Scc4 homologs in yeasts and animals with residues mutated in the *scc4*-*m3* allele shown. (B) Chromatin association of *X. laevis* Scc2^1–1024^-Scc4 depends on the Scc4 conserved patch. *X. laevis* high-speed supernatant (HSS) was incubated with sperm chromatin and recombinant Scc2^1–1024^-Scc4. Chromatin-associated proteins were recovered by sucrose sedimentation and detected by autoradiography (Scc2^1–1024^-Scc4) or western blot (Orc2; ^∗^non-specific band). (C) Schematic showing idealized kinase activity during the cell cycle. (D) Cartoon comparing DDK-dependent targeted cohesin loading at the yeast centromere with a similar process in vertebrates. See also Figure S6.

**Figure S6:**
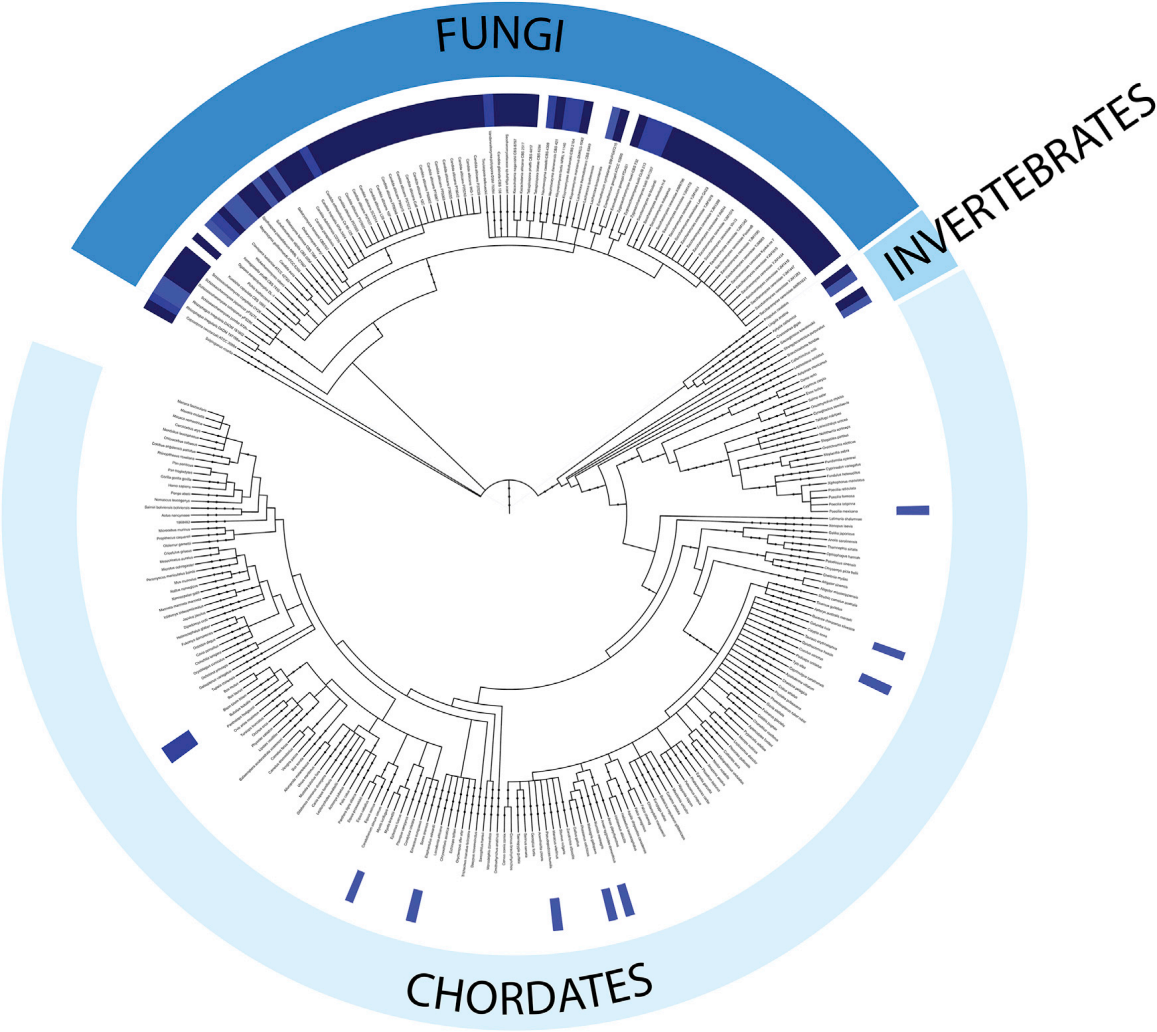
Phylogenetic Analysis of Candidate Ctf19 Phosphorylation Sites, Related to Figure 7 Candidate Ctf19 phosphorylation sites are widespread in yeasts and largely absent from chordates. N-terminal fragments of Ctf19 homologs for all listed species were examined for the presence of adjacent serine/threonine residues (SS, ST, TS, or TT). Blue boxes immediately surrounding the species names indicate the presence of candidate sites, with darker blue corresponding to multiple sites.

The mechanism of Scc2/4 targeting we have described depends on a surface of Scc4 conserved from yeasts to vertebrates (Figure 7A). Ctf19 phosphorylation sites are broadly conserved in yeasts, but we found that vertebrate Ctf19 homologs, including *X. laevis* CENP-P, do not have candidate phosphorylation sites that align well with those required for Scc2 recruitment in yeast (Figure S6). DDK activity is nonetheless required for Scc2/4 to localize to chromatin in vertebrates (Takahashi et al., 2008). To test whether the Scc4 conserved patch is required for vertebrate Scc2/4 recruitment, we tested whether *X. laevis* Scc2^1–1024^-Mau2^Scc4^ can associate with chromatin upon incubation with high speed egg supernatant (HSS; Figure 7B). We found that a version of Mau2^Scc4^ that mimics the yeast *scc4*-*m3* mutant (Mau2^Scc4^ Y307A, K310D, K331A; m3) did not associate with chromatin. We conclude that a mechanism analogous to the one we have described for Scc2/4 localization in yeast is also present in vertebrates.

## Discussion

We have presented a molecular explanation for targeted cohesin loading at centromeres. The following requirements for this pathway have been demonstrated previously: (1) kinetochore assembly and, more specifically, the Ctf19 complex (Eckert et al., 2007, Fernius and Marston, 2009, Ng et al., 2009), (2) the activity and centromeric localization of DDK (Natsume et al., 2013), (3) a conserved structural feature of the Scc4 protein (Hinshaw et al., 2015), and (4) expression of the Scc1 protein (Fernius et al., 2013). We have determined that phosphorylation of Ctf19 by DDK provides a binding site for Scc4 at kinetochores, explaining the first three of these four requirements.

These findings support a two-step process for cohesin recruitment to centromeres. First, the Ctf19 complex, through Ctf3, recruits DDK in G1, which phosphorylates the Ctf19 protein and possibly other targets. Second, phosphorylated Ctf19 recruits the cohesin loader. Centromere cohesion requires both steps, while early replication of centromeric DNA, requires only the first (Natsume et al., 2013). Our identification of separation of function mutations that do not affect DDK localization and activity but that do perturb cohesin localization to centromeres strongly supports the two-step model. Furthermore, deletion of factors important for DDK recruitment (*chl4*Δ or *ctf3*Δ) does not perturb Ctf19 localization at centromeres (Natsume et al., 2013, Pot et al., 2003), indicating that failure to phosphorylate Ctf19 is what prevents Scc2/4 recruitment. This division of labor between Ctf19 complex components—one acts as the DDK recruiter (Ctf3) and another acts as the DDK substrate (Ctf19)—was confirmed by our reconstitution of centromere-targeted cohesin loading in vivo by tethering DDK in the absence of the recruiter.

The first step in centromeric cohesin enrichment is specification of centromeres as preferred loading sites. Several lines of evidence have established that a Ctf19 complex-dependent pathway operates before S phase to achieve this (Figure 7C). First, DDK appears at centromeres before DNA replication (Natsume et al., 2013)(Figures 3, S4A, and S4B), and low levels of Ctf19 phosphorylation are apparent in G1 (Figures 2C and S5A). Second, overexpression of the limiting cohesin subunit, Scc1, drives Scc2 onto centromeres in G1 and depends on the Ctf19 complex to do so (Fernius et al., 2013). Third, vertebrate Scc2/4 associates with chromatin before DNA replication (Figure 7B) (Takahashi et al., 2008). The same is probably also true of yeast Scc2/4: (1) depletion of the DNA replication factor, Cdc6, does not prevent cohesin loading (Fernius et al., 2013), and (2) cells arrested in late G1 by expression of a dominant allele of the Cdc28/Cdk1 inhibitor Sic1 have a cohesin distribution identical to that seen in metaphase (Lopez-Serra et al., 2013).

The second step in centromeric cohesin enrichment is the specific docking of Scc2/4 onto phosphorylated Ctf19 at kinetochores. The factors we have described here are sufficient for this activity in vitro (Figure 2A), but Scc1 expression is required in vivo. Scc1 production at cell-cycle entry completes the cohesin ring and enables its association with the Scc2/4 cohesin loader (Fernius et al., 2013). Therefore, cohesin potentiates the ability of Scc2/4 to associate with phosphorylated Ctf19 by a yet undetermined mechanism.

Cohesin remains on chromatin and is activated for cohesion during S phase (Rhodes et al., 2017, Uhlmann and Nasmyth, 1998). The two-step pathway we have described ensures that cohesin is efficiently loaded onto centromeres before they replicate, consistent with the observation that slowing DNA replication partially ameliorates chromosome segregation defects in Ctf19 complex mutants (Fernius and Marston, 2009). Moreover, our observation that phosphorylated Ctf19 accumulates in *chl4*Δ strains upon DDK overproduction suggests that Chl4 and Ctf3 amplify DDK’s activity locally when DDK levels would otherwise be limiting. In this way, when Dbf4 levels begin to rise in late G1 (Jackson et al., 1993), the centromere is assured both of replicating early and of accumulating high levels of cohesin (Figure 7C).

We have previously proposed the existence of two modes of cohesin loading (Hinshaw et al., 2015). One mode operates specifically at centromeres and depends on the Scc4 conserved patch and the factors we have described here. A second mode results in cohesin loading genome-wide and is sufficient to confer viability. How Scc2/4 and cohesin are recruited to chromatin in the second mode is still unclear but may involve the RSC chromatin remodeling complex (Lopez-Serra et al., 2014).

How is Scc4 localization regulated in other chromosomal contexts? We propose an analogous process, in which phosphorylated chromosomal factors other than Ctf19 recruit Scc2/4. In support of this idea, the Scc4 conserved patch mutant (*scc4*-*m35*) displays meiotic cohesin localization defects on chromosome arms (Vincenten et al., 2015), hinting at the existence of meiosis-specific Scc4 binding partners. Furthermore, a DDK- and Scc4-dependent Scc2/4 localization pathway operates in *X. laevis* egg extracts (Takahashi et al., 2008), although the DDK substrate in this pathway is not known (Figure 7D). It is therefore likely that the mechanism we have described here reflects general principles in cohesin-mediated chromosome organization.

## STAR★Methods

### Key Resources Table

**Table:** 

REAGENT or RESOURCE	SOURCE	IDENTIFIER
**Antibodies**		

Mouse monocolonal anti-FLAG-M2-HRP	Sigma	Cat#A8592; RRID: AB_439702
Mouse monoclonal anti-GFP	Roche	Cat#11814460001; RRID: AB_390913
Rabbit polyclonal anti-PGK1	Marston lab stock	N/A
Mouse monoclonal anti-myc 9E10	Sigma	Cat#M4439; RRID: AB_439694
Rabbit polyclonal anti-6His-HRP	Abcam	Cat#ab1187; RRID: AB_298652
Mouse monoclonal anti-HA 12CA5	Roche	Cat#11666606001
Rabbit polyclonal anti-xORC2	Generous gift from Thomas Graham, Laboratory of Johannes Walter; (Takahashi et al., 2008)	N/A

**Bacterial and Virus Strains**		

*E. coli* Rosetta 2(DE3)pLysS	EMD Millipore	Cat#71403

**Biological Samples**		

*Xenopus laevis* high speed egg supernatant	Generous gift from Thomas Graham, Laboratory of Johannes Walter	N/A
*Xenopus laevis* sperm chromatin	Generous gift from Thomas Graham, Laboratory of Johannes Walter	N/A

**Chemicals, Peptides, and Recombinant Proteins**		

MDFpTpSD	Tufts Univ. Core Facility – Peptide Synthesis	N/A
MDFTSDTTNSH	Tufts Univ. Core Facility – Peptide Synthesis	N/A
MDFpTSDTTNSH	Tufts Univ. Core Facility – Peptide Synthesis	N/A
MDFTpSDTTNSH	Tufts Univ. Core Facility – Peptide Synthesis	N/A
ATP, [γ-^32^P]	Perkin-Elmer	Cat#BLU002A
EasyTag L-[^35^S]-Methionine	Perkin-Elmer	Cat#NEG709A
Concanavalin A fron *Canavalia ensiformis*	Sigma	Cat#C5275
Phostag Acrylamide	Wako	Cat#300-93523 (AAL-107M)
Anti-FLAG-M2 Magnetic Beads	Sigma	Cat#M8823
IgG Sepharose 6 Fast Flow	GE Life Sciences	Cat#17-0969-01
PP1 Analog	EMD Millipore	Cat#529579
Dynabeads Protein G	Life Technologies	Cat#1000D
Rapamycin	Cambridge BioScience	Cat#R001
Chelex 100 Resin	BioRad	Cat#1421253
Express SYBR Green	Invitrogen	Cat#1000011652
Zirconia/Silica Beads	BioSpec Products	Cat#110791052

**Deposited Data**		

Structure factors and atomic coordinates for Scc2/4 in complex with Ctf19 peptide	This study	PDB: 5W94

**Experimental Models: Organisms/Strains**		

See Tables S4 and S5 for yeast strains used in this study	N/A	N/A

**Recombinant DNA**		

pSMH1079: pGAL1-10::FLAG-Cdc7-AS3-CBP-TEV-proA:Dbf4; LEU2	This study	N/A
pSMH1080: pGAL1-10::FLAG-Cdc7-AS3-CBP-TEV-proA:Dbf4; LEU2	This study	N/A
pSMH7: pT7-6His-GST (LIC)	Berkeley Q3B	Cat#29655 (Addgene)
pSMH30: pT7-6His-GST-Chl4; 6His-Iml3	(Hinshaw and Harrison, 2013)	N/A
pSMH79: pT7-6His-GST-Chl4^1-445^; 6His-Iml3	(Hinshaw and Harrison, 2013)	N/A
pSMH12: pT7-6His-Iml3	(Hinshaw and Harrison, 2013)	N/A
pSMH145: pT7-6His-Ctf3; 6His-Mcm16; 6His-Mcm22	This study	N/A
pSMH154: pT7-6His-GST-Mcm16; 6His-Mcm22	This study	N/A
pSMH74: pFS1 (pT7-Ctf19; 6His-Mcm21)	This study	N/A
pSMH1004: pT7-Ctf19-Δ30; 6His-Mcm21	This study	N/A
pSMH1003: pT7-Ctf19-9A; 6His-Mcm21	This study	N/A
pSMH1002: pT7-Ctf19-3A-1; 6His-Mcm21	This study	N/A
pSMH992: pT7-6His-MBP-Ctf19; His6-Mcm21	This study	N/A
pSMH1084: pT7-Ctf19-6A; 6His-Mcm21	This study	N/A
pSMH1085: pT7-Ctf19-7A; 6His-Mcm21	This study	N/A
pSMH1086: pT7-Ctf19-2A; 6His-Mcm21	This study	N/A
pSMH1046: pT7-Kozak-Dbf4 (IVT)	This study	N/A
pSMH1047: pT7- Kozak-Cdc7 (IVT)	This study	N/A
pSMH716: pT7- Kozak-xMau2 (IVT)	This study	N/A
pSMH717: pT7- Kozak-xMau2 m3 (IVT)	This study	N/A
pSMH703: pTT220 (pT7-S-tag-xScc2N) (IVT)	(Takahashi et al., 2008)	N/A
pSMH685: pT7-6His-FLAG-Scc2n; Scc4	This study	N/A
pSMH686: pT7-6-His-FLAG-Scc2n; Scc4-m3	This study	N/A
pSMH719: pT7-6His-Ninl (lambda phosphatase)	This study	N/A
pSMH9: pRK793 (pT7-His-TEV)	(Tropea et al., 2009)	Cat#8827 (Addgene)

**Software and Algorithms**		

MetaMorph Acquisition Software	Molecular Devices	https://www.moleculardevices.com/systems/metamorph-research-imaging/
ImageJ	National Institutes of Health	https://imagej.nih.gov/ij/
Prism 7	GraphPad	https://graphpad.com
Mafft	(Katoh et al., 2002)	http://mafft.cbrc.jp/alignment/software/
JalView	(Waterhouse et al., 2009)	http://www.jalview.org/
phyloT	Biobyte	http://phylot.biobyte.de/
IToL	(Letunic and Bork, 2007)	http://itol.embl.de/
HKL-2000	(Otwinowski and Minor, 1997)	http://www.hkl-xray.com/
Phaser MR	(McCoy et al., 2007)	http://www.ccp4.ac.uk/
DM (DensityModification module)	CCP4	http://www.ccp4.ac.uk/html/dm.html
PHENIX	(Adams et al., 2010)	https://www.phenix-online.org/
MATLAB	MathWorks	https://www.mathworks.com/
COOT	(Emsley et al., 2010)	https://www2.mrc-lmb.cam.ac.uk/personal/pemsley/coot/

### Contacts for Reagent and Resource Sharing

Further information and requests for resources and reagents should be directed to and will be fulfilled by Lead Contact, Stephen C. Harrison (harrison@crystal.harvard.edu).

### Experimental Model and Subject Details

All yeast strains were derivatives of w303 or s288c and grown at 30°C or room temperature. Other details of growth conditions are given in the figure legends. Nocodazole was used at 15 μg/ml, benomyl at 30 μg/ml and rapamycin at 1 μM.

### Method Details

#### Recombinant protein production

6His-tagged recombinant Ctf19 complex proteins were expressed in *E. coli* and purified as described previously (Hinshaw and Harrison, 2013). With the exception of Mif2^256-566^, each recombinant protein preparation was subjected to size exclusion chromatography (Superdex 200, GE) in GF200 (20mM Tris-HCl, pH 8.5; 200mM NaCl; 1mM tris(2-carboxyelthyl)phosphine) as a final step. Mif2^256-566^ was frozen for permanent storage immediately following ion exchange chromatography (HiTrap Q HP, GE). All recombinant samples were frozen in purification buffer supplemented with 5% glycerol (v/v), and stored at −80°C until use. In cases where two or more proteins are members of the same complex, we created and used vectors coding for all complex subunits on a single RNA transcript, each with its own ribosome binding site.

For crystallography, the N-terminal tags were cleaved from Scc2^1-181^-Scc4 by exposure to TEV protease for two hours at room temperature followed by tag removal by Ni-NTA chromatography. Following this step, cleaved Scc2^1-181^-Scc4 was further purified by size exclusion chromatography, and peak fractions were concentrated by ultrafiltration to a final concentration of ∼500μM.

#### In vitro translation and pulldowns

In vitro translation of DDK subunits was performed using an equimolar mix of two plasmids, one encoding Dbf4 and one encoding Cdc7, as templates for transcription and translation according to the manufacturer’s instructions (Promega). 10μl of translation products were used for each pulldown reaction. Radiolabeled DDK translation mix was added to equal molar amounts of each recombinant His-tagged protein complex. Mixtures were incubated on ice for one hour, and pulldowns and analysis were performed as described previously (Hinshaw and Harrison, 2013). Briefly, NiNTA-coupled beads (QIAGEN) were equilibrated in pulldown buffer (30mM Tris, pH 8; 30mM imidazole, pH 8; 150mM NaCl; 0.05% NP-40 substitute; 10% glycerol; 2mM β-mercaptoethanol). After end-over-end incubation for one hour, beads were pelleted by centrifugation and washed three times with pulldown buffer. Proteins were released from beads with SDS-PAGE loading buffer and analyzed by SDS-PAGE and autoradiography.

#### DDK purification

An *S. cerevisiae* strain in which Cdc7 has been tagged at its C terminus with a TAP tag (calmodulin binding peptide – TEV site – tandem Protein A tags) was grown to an optical density (A600) of 2 in 12 l of yeast extract peptone dextrose (YPD) medium. For experiments involving analog-sensitive DDK, we created an *S. cerevisiae* strain bearing FLAG-Cdc7^AS3^-CBP-TEV-ProA (with only a single Protein A tag) and untagged Dbf4, both under the control of a bidirectional galactose-inducible promoter and integrated at *LEU2*. Cells were grown in 4 to 8 l of yeast extract peptone (YP) medium with 2% raffinose (w/v) to A600 0.8. DDK expression was induced by addition of 1:10 (v/v) 20% galactose, and cells were grown for four hours longer. All growths and expressions were done at 30°C. Identical procedures were carried out for kinase purifications in both cases.

Cells were pelleted, washed once in PBS with 1mM PMSF, and resuspended at a 1:1 ratio (w/v) in buffer W400+ (400mM NaCl; 50mM HEPES, pH 7.5; 10% (v/v) glycerol; 1mM EDTA; 0.05% (v/v) NP-40 substitute; 0.5mM PMSF; 4μM aprotinin; 1μM leupeptin; 1.4μM pepstatin; 20mM NaF; 2mM NaoVO_4_; 0.02% Na-azide). Cell slurry was flash frozen in liquid nitrogen, and frozen cell pellets were pulverized using a Retsch ball mill with liquid nitrogen-cooled canisters. Cell powder was stored at −80°C until purification.

For purification, yeast powder was thawed, and buffer W400+ was added at a 1:1 (v/v) ratio. After homogenization by sonication and addition of RNase A (Sigma) to a final concentration of 10μg/ml, lysate was subjected to two rounds of centrifugation after which the supernatant was collected each time (1 × 10 min and 1 × 30 min, both at 18,000 rpm in a Beckman JA-20 rotor). Cleared lysates were incubated for two hours with 1.5ml IgG Sepharose fast flow resin (GE) before washing at 1.7ml/min with 50ml ice-cold W400+. Protease inhibitors were then removed by washing with 25ml of ice-cold W400- (equivalent to W400+ without PMSF, aprotinin, leupeptin, and pepstatin). Resin was then resuspended in 10ml W400- and incubated for four hours in the presence of TEV protease at 4°C. Proteins released from the resin were recovered, and the resin was washed with 40ml ice-cold W25+ (W400+ with 25mM NaCl). The pooled eluate was mixed and applied to a 5ml cation exchange column (HiTrap SP HP, GE). Fractions were collected across an eight column volume gradient from W25+ to W1000+ (W400+ with 1M NaCl). Eluted fractions were analyzed by SDS-PAGE and silver staining, and DDK-containing fractions were pooled, concentrated, and subjected to size exclusion chromatography (Superdex 200, GE) in GF300+ (300mM NaCl; 50mM HEPES, pH 7.5; 1mM EDTA; 0.5mM PMSF; 1mM DTT). Gel filtration fractions were analyzed as for anion exchange fractions, and DDK-containing fractions were pooled and analyzed for autocatalytic activity by radioactive phosphate transfer as described below.

#### DDK activity assay

For phosphorylation reactions, purified DDK was concentrated to approximately 20uM as determined by absorbance at 280nm. Each phosphorylation reaction contained 4μL purified DDK, 10μL of substrate protein (1.8μM final concentration), 1μL ATP mix (100μM ATP-MgCl_2_ supplemented with a final concentration of 0.5μCi γ-^32^[P]-ATP per reaction), and 5μL 2x DDK reaction buffer (100mM HEPES, pH 7.5; 10mM Mg(OAc)_2_, 20% (v/v) glycerol, 400mM potassium glutamate, 2mM EDTA, 0.02% (v/v) NP-40 substitute; 4mM NaoVO_4_; 40mM NaF; 2mM β-mercaptoethanol). Reactions were incubated for one hour at 30°C and stopped by the addition of SDS-PAGE loading buffer, followed by boiling to denature all proteins. These reaction conditions were designed to closely mimic those previously reported by the Bell laboratory (Francis et al., 2009). Reaction products were analyzed by SDS-PAGE and autoradiography.

#### Peptide binding experiments

We used biolayer interferometry (ForteBio) to quantify the interaction between Scc2^1-181^–Scc4 and synthetic peptides derived from the N-terminal region of Ctf19 (Tufts University Core Facility, Boston). For all steps, we used octet buffer (40mM HEPES, pH 7.5; 200mM NaCl; 1mM DTT) and low-volume 96-well opaque black plates. Purified Scc2^1-181^-Scc4 (in which both proteins had N-terminal 6His tags and Scc2^1-181^ had a single FLAG tag following the His tag) was captured on NiNTA fiber-optic tips for 30 s, followed by equilibration in buffer and subsequent incubation with Ctf19 peptides at varying concentrations. This step was allowed to reach saturation before a final incubation in octet buffer.

To derive binding curves, we first applied a linear correction so that all association steps began at zero response units. We then performed a linear drift correction by subtracting the signal for Scc2^1-181^-Scc4 incubated in buffer alone during the association step (no peptide, linear subtraction for each time point, independent control for each experiment). We then applied a loading factor to correct for the Scc2^1-181^-Scc4 immobilization efficiency for each well. From these normalized association curves, we extracted maximum responses for each well by averaging the signal across the final ten seconds of the association step. We performed these operations for three independent experiments, performed with new tips and samples, for each data point. Peptides incubated at the highest concentrations tested with tips alone did not produce detectable binding. Data points represent mean maximum responses, and error bars show the standard deviation for the three experiments. Fits were determined using GraphPad Prism software assuming a single specific binding site.

#### Crystal preparation

Scc2^1-181^-Scc4 complex was prepared as described above. A synthetic Ctf19^1-6^ diphosphorylated peptide was mixed with TEV-cleaved Scc2^1-181^-Scc4 at a molar ratio of 1:1.8 (Scc2^1-181^-Scc4 complex:peptide) and a final concentration of 25mg/ml (total protein). After incubation for one hour at 4°C, the sample was mixed at a 1:1 ratio (v/v) with crystallization buffer (8% PEG 4,000; 80mM PIPES, pH 6) and incubated at 18°C in a sitting drop crystallization setup. Crystals appeared within two days and were harvested after seven days. Crystals were incubated briefly in crystallization buffer containing 30% glycerol (v/v) before flash freezing in liquid nitrogen.

#### Structure determination

Data were collected at beamline 24-IDE at the Advanced Photon Source, Argonne National Laboratory. Data were merged and scaled using HKL-2000 (Otwinowski and Minor, 1997). Crystals diffracted to a minimum Bragg spacing of 3.19Å and conformed to the space group P6_2_. Initial phase determination was achieved by molecular replacement using trimmed Scc2^1-181^-Scc4 coordinates (PDB: 4XDN with affinity tag residues removed) as a search model and the Phaser MR module as implemented by CCP4 (McCoy et al., 2007). In our search, we specified two subunits per asymmetric unit of the crystal. After generation of a non-crystallographic symmetry (NCS) density mask using the ncsmask program and the starting coordinates, ten rounds of phase improvement by non-crystallographic symmetry (NCS) averaging with density modification were carried out using the DM module in CCP4. Inspection of the resulting electron density maps revealed unaccounted-for density at the surface of each Scc4 molecule. Minimal peptide fragments were built into this density in *Coot* (Emsley et al., 2010), and the resulting model was subjected to iterative refinement using Phenix Refine (Adams et al., 2010) with NCS taken into account.

#### Strain construction and growth conditions

All mutations were introduced by integration of PCR products containing the mutation of interest and a selectable marker according to standard techniques (Longtine et al., 1998). Strains for sister centromere cohesion assays were generated by mating Ctf19 mutant strains to the centromere tester strain and subsequent sporulation to recover haploids. Each marker was checked independently.

For cell cycle arrests not detailed in figure legends, cultures were resuspended in fresh medium containing 5μg/ml alpha factor (Genescript), 200mM hydroxyurea (Sigma), or 150μg/ml nocodazole and 10μg/ml benomyl. Alpha factor and nocodazole were re-added after one hour to appropriate cultures. Cells were harvested two hours after initiating the arrests.

#### Cohesion assay

Chromosome dot assays were performed essentially as described (Fernius and Marston, 2009). For imaging, cells were fixed with 10% (v/v) formalin before resuspension in phosphate buffered saline (PBS) supplemented with 1.2M sorbitol. Cells were immobilized on coverslips coated with concanavalin-A (Sigma) and imaged using a Nikon TiE motorized inverted microscope with a Nikon Plan Apo 60x objective lens (NA 1.4) and a Hamamatsu ORCA-R2 cooled digital camera. Z stacks (.35μm spacing) from multiple stage positions for each sample were acquired using the Perfect Focus System and MetaMorph software. Maximum projections along the Z axis were generated and analyzed in ImageJ.

#### Artificial tethering of Dbf4

To artificially tether Dbf4 to the kinetochores, Dbf4 was tagged with FRB (pFA6a-FRB-KanMX6), and *CTF19*, *ctf19-9A* and *ctf19-2A* were tagged with FKBP12-TRP1 (pFA6a-FKBP12-TRP1). Proteins were tagged at their C-termini by one-step cassette integrations at their original gene loci as previously described (Haruki et al., 2008). Yeast strains for these experiments also carried *TOR1-1*, which confers rapamycin resistance, and the *fpr1Δ* mutation. Tethering of FRB and FKBP12 fusion proteins was induced by addition of rapamycin to the medium at a final concentration of 1 μM. For control cultures, an equal volume of DMSO was added instead.

#### Live-cell GFP fusion protein imaging

Cells were grown overnight in synthetic complete medium and imaging cultures were inoculated the next morning in new medium at a 1:40 dilution (v/v). Cells were grown for 6-8 hr before imaging using the setup described above. Live cells adhered to concanavalin-A-coated coverslips were washed in fresh medium immediately before mounting in a Tokai Hit stage-top incubator set to 30°C. For Scc2-GFP experiments, exposure times were as follows: DIC – 20ms, tdTomato – 7ms, GFP – 250ms. For Cdc7-GFP experiments, exposure times were as follows: DIC – 20ms, mCherry – 25ms, GFP – 200ms. For Sld7-GFP experiments, exposure times were as follows: DIC – 20ms, mCherry – 50ms, GFP – 250ms. For Scc2-GFP and Cdc7-GFP experiments, images were captured every 10 min, with eight or nine focal planes (.35μm spacing) collected for each stage position and color. A single time point was collected for Sld7-GFP cells arrested in G1 with alpha factor. While we were able to observe Sld7-GFP foci in alpha factor-arrested cells, Cdc7-GFP foci were only clearly visible in cycling cells immediately prior to S-phase. This observation and magnitude of the effect of *CTF19* deletion on Sld7 and Cdc7 localization agree well with previously published observations (Natsume et al., 2013).

Four stage positions were imaged for each strain, and this procedure was repeated for at least three independent cultures for each strain. Data from all stage positions were pooled for each experiment, and statistics (means and standard deviations reported in figures) represent summaries of all individual experiments (conducted on at least three separate occasions for each strain). Image processing and analysis was performed in ImageJ. Figures were prepared in Photoshop (Adobe). For figure preparation, identical processing steps were applied to maximum projections along the Z axis (Scc2-GFP strains) or to single focal planes (Cdc7-GFP and Sld7-GFP) for all images in each panel.

#### Chromatin immunoprecipitation

ChIP using anti-HA (12CA5, Roche), anti-FLAG (M2, Sigma) or anti-GFP (Roche) was performed as previously described (Hinshaw et al., 2015). Briefly, cells were fixed in 1% formaldehyde, washed, resuspended in ice-cold FA lysis buffer (50mM HEPES-KOH at pH 7.5, 150mM NaCl, 1mM EDTA, 1% v/v Triton X-100, 0.1% w/v Sodium Deoxycholate, 0.1% w/v SDS), and lysed in a Fastprep Bio-pulverizer FP120 with silica beads (Biospec Products). Samples were sonicated to fragment chromosomal DNA using a BioRupter (Diagenode). Aliquots of the resultant chromatin solution were incubated with antibody and Dynabeads Protein G (Life Technologies) overnight at 4°C. Following sequential washes, immunoprecipitated and 1/100 input chromatin was recovered by boiling (10 min) with a 10% slurry of Chelex-100 resin before adding Proteinase K (0.125 mg) and incubating at 55°C for 30 min. Samples were boiled again, centrifuged and the supernatant extracted for qPCR analysis on a Roche Lightcycler using Express SYBR green reagent (Invitrogen). Primers used for qPCR analysis are previously described (Hinshaw et al., 2015). To calculate ChIP enrichment/input, ΔCT was calculated according to: ΔCT = (CT_(ChIP)_ − [CT_(Input)_− logE (Input dilution factor)]) where E represents the specific primer efficiency value. Enrichment/input value was obtained from the following formula: E^−ΔCT^. qPCR was performed in triplicate, typically for each of three or more independent cultures. Error bars represent standard error.

#### Western blotting

Antibodies for western blotting are listed in the Key Resources Table. Mouse anti-HA (12CA5, Roche), mouse anti-FLAG (Mono M2, Sigma Aldrich, St Louis, Missouri), mouse anti-GFP (Roche), and rabbit anti-6His-HRP (Abcam) were used at a dilution of 1:1000. Rabbit anti-Pgk1 (Marston lab stock) was used at a dilution of 1:10000.

#### In vivo Ctf19 phosphorylation assay

For each sample, a 50mL culture was maintained in logarithmic growth phase overnight. Cells were harvested or arrested in mid-log phase (A600 0.5-1.0). After harvesting, cells were washed once in cold PBS supplemented with 80mM NaF, 20mM NaoVO_4_, and 80mM β-glycerol phosphate. Cell cycle distributions were confirmed visually and by flow cytometry for all experiments involving arrests. Pellets were flash-frozen in liquid nitrogen and stored at −80°C until sample preparation. Cells were lysed by bead beating with 0.5mm glass beads (Biospec) in 400μL lysis buffer (40mm HEPES, pH 7.5; 400mM NaCl; 10% glycerol (v/v); 0.1% NP-40 substitute (v/v); 80mM NaF; 20mM NaoVO_4_; 80mM β-glycerol phosphate; 1mM PMSF; 40μM aprotinin; 10μM leupeptin; 14μM pepstatin). Lysates were cleared by centrifugation at 14,000 rpm in a microcentrifuge for 10 min at 4°C. Soluble material was first cleared by gentle rotation for one hour with glutathione Sepharose resin (GE) at 4°C. Cleared lysate was then incubated for two hours with anti-FLAG-M2 magnetic resin (Sigma) at 4°C with rotation. Magnetic beads were washed four times with wash buffer (lysis buffer with 2% Triton X-100 added) before elution of bound proteins by addition of 2x Laemmli SDS-PAGE sample buffer and boiling. For analysis of Ctf19 phosphorylation, samples were resolved on Zn^2+^-Phostag PAGE gels containing 10μM phostag reagent (Waco) and 9.6% acrylamide/bis at neutral pH according to the manufacturer’s recommendations. Separated proteins were transferred to PVDF membranes and probed by western blot (anti-FLAG-M2-HRP; Sigma).

#### Chromatin association in *X. laevis* HSS

Individual i*n vitro* transcription and translation reactions for each of the three recombinant proteins were set up as described above. Reactions were flash-frozen in liquid nitrogen and stored at −80°C until use. Before freezing and use in chromatin association experiments, we normalized input concentrations by resolving aliquots on SDS-PAGE gels and quantifying band intensities by autoradiography. Prior to incubation with chromatin, inputs were incubated with each other (Scc2^1-1024^ with Scc4^Mau2-WT^ or Scc4^Mau2-m3^) for one hour on ice. Chromatin association experiments were performed as described previously (Takahashi et al., 2008). *X. laevis* sperm chromatin was activated by exposure to high speed supernatant (HSS) for 40 min at room temperature. Recombinant Scc2^1-1024^-Scc4 was then added, and the reaction was incubated 10 min further before pelleting and washing of chromatin. Input and pelleted samples were resolved by SDS-PAGE and visualized by autoradiography. The experiment was repeated three times. Orc2 antibody was a gift from the laboratory of Johannes Walter (Harvard Medical School).

#### Alignments and evolutionary conservation analysis

Multiple sequence alignments were compiled using NCBI PSI-BLAST. Alignments were generated using MAFFT and visualized with Jalview (Katoh et al., 2002, Waterhouse et al., 2009). For phylogenetic tree construction and candidate phosphorylation site analysis, we used custom Python scripts to parse the N-terminal sequences of all unique CENP-P/Ctf19 sequences shown (Figure S6). The tree was generated using phyloT and visualized using the Interactive Tree of Life (Letunic and Bork, 2007). We also examined the phylogenetic distribution of CENP-P homologs with at least two S/T pairs (SS, ST, TS, or TT) mapping to the flexible N-terminal region and observed a result that was in close qualitative agreement with the one presented in Figure S6.

### Quantification and Statistical Analysis

Conventions used to indicate statistical comparisons between conditions and the associated statistical tests are described in figure legends for the corresponding comparisons. In cases where multiple biological measurements were made (especially imaging and ChIP experiments), at least three independent cultures of the indicated strain contribute to each reported value. For all imaging experiments, at least three different genotypes were imaged on the same slide during each imaging session, and a wild-type strain was always included to ensure quantitative comparability with previous observations (to ensure microscope, camera, growth chamber, and growth conditions remained uniform across replicates).

### Data and Software Availability

The accession number for the atomic coordinates and structure factors corresponding to the Scc2/4 complex bound to the Ctf19 phosphorylated peptide reported in this paper is PDB: 5W94.

## Author Contributions

All authors contributed to the conception and design of the project. All authors participated in writing the manuscript. S.M.H. and V.M. performed experiments. S.M.H., V.M., and A.L.M. generated reagents.
