# Supplementary material for: The Kinetochore Receptor for the Cohesin Loading Complex
Source: Cell. 2017 Sep 21;171(1):72–84.e13. doi: 10.1016/j.cell.2017.08.017 (PMC5610175; doi:10.1016/j.cell.2017.08.017)
Supplement: Document S1. Tables S1–S5 [file mmc1.pdf]

Cell, Volume *171*

Supplemental Information

The Kinetochore Receptor for the Cohesin Loading Complex

Stephen M. Hinshaw, Vasso Makrantoni, Stephen C. Harrison, and Adèle L. Marston

Supplemental Tables

Table S1. Related to Figure 1 – *CTF19* alleles used in this work

Name	Mutations
ctf19-2A	*T4A, S5A*
ctf19-3A-1	*S5A, T8A, S14A*
ctf19-3A-2	*T4A, S5A, T7A*
ctf19-6A	*T8A, S10A, T13A, S14A, S16A, S19A*
ctf19-7A	*T7A, T8A, S10A, T13A, S14A, S16A, S19A*
ctf19-9A	*T4A, S5A, T7A, T8A, S10A, T13A, S14A, S16A, S19A*
ctf19-2D	*T4D, S5D*
ctf19-2E	*T4E, S5E*

Table S2. Related to Figure 6 – *SCC4* alleles used or discussed in this work

Name	Mutations
scc4-m3	*F324A, K327D, K331D*
scc4-m35	*F324A, K327D, K331D, K540A, K541A*
scc4-m7	*L256K, Y298A, K299D, Y313A, F324A, K327D, K331D*

Table S3. Related to Figure 6 – Crystallography statistics

**Data Collection**	$Scc2^{1\text{-}181}$-Scc4-$Ctf19^{1\text{-}6}$
Resolution (Å)	39.94-3.19
Wavelength (Å)	0.97918
Space Group	$P6_2$
Unit Cell Dimensions (a, b, c) (Å)	173.0 173.0 145.1
Unit Cell Angles (α , β , γ) (Å)	90 90 120
I/ σ (last shell)	24.37 (1.0)
R_{sym} (last shell) (%)	5.5 (106)
Completeness (last shell) (%)	99.11 (97.9)
Redundancy (last shell)	10.6 (10.3)
Unique Reflections (last shell)	40688 (2026)
**Refinement**	
Resolution (Å)	39.94-3.19
Number of Reflections	40629
working	38633
free	1994
R_{work} (last shell) (%)	20.9 (37.2)
R_{free} (last shell) (%)	25.8 (41.6)
**Structure Statistics**	
r.m.s.d. bond lengths	0.011
r.m.s.d. bond angles	1.42

Table S4. Related to STAR Methods – Yeast strains built in w303

Strain Number	Genotype	Reference
AM1145	*MATa SCC1-6HA*	Fernius et al., 2013
AM1176	*MATa* W303 wild-type	Fernius et al., 2013
AM4643	*MATa pMET-CDC20 2.4CEN4*(2.4 kb right of *CEN4*)*::tetOs tetR-GFP SPC42-tdTomato*	Fernius et al., 2013
AM6006	*MATa SCC2-6HIS-3FLAG::KanMX6*	Fernius et al., 2013
AM20613	*MATa SCC2-6HIS-3FLAG::KanMX CTF19::HIS3*	This study
AM20619	*MATa SCC2-6HIS-3FLAG::KanMX ctf19-9A::HIS3*	This study
AM20622	*MATa SCC2-6HIS-3FLAG::KanMX ctf19-6A::HIS3*	This study
AM20629	*MATa SCC1-6HA CTF19::HIS3*	This study
AM21110	*MATa CDC7-6HIS-3FLAG::URA3*	This study
AM21234	*MATa CDC7-6HIS-3FLAG::URA3 ctf19-9A::HIS3*	This study
AM21486	*MATa tor1-1 fpr1::NAT DBF4-FRB::KanMX CTF19-FKBP12::TRP1*	This study
AM21613	*MATa tor1-1 fpr1::NAT DBF4-FRB::KanMX CTF19-FKBP12::TRP1 SCC2-6HIS-3FLAG::KanMX*	This study
AM21614	*MATa tor1-1 fpr1::NAT DBF4-FRB::KanMX CTF19-FKBP12::TRP1 SCC2-6HIS-3FLAG::KanMX ctf3Δ::KanMX*	This study
AM21617	*MATa tor1-1 fpr1::NAT DBF4-FRB::KanMX ctf19-9A-FKBP12::TRP1 SCC2-6HIS-3FLAG::KanMX ctf3Δ::KanMX*	This study
AM21681	*MATa tor1-1 fpr1::NAT DBF4-FRB::KanMX ctf19-9A-FKBP12::TRP1 SCC2-6HIS-3FLAG::KanMX*	This study
AM21871	*MATa 3yGFP-DBF4*	This study
AM21872	*MATa 3yGFP-DBF4 ctf19-9A::HIS3*	This study

AM21908	*MATa SCC2-6HIS-3FLAG::KanMX ctf19-2A::HIS3*	This study
AM21958	*MATa SCC1-6HA ctf19-2A::HIS3*	This study
AM22083	*MATa pMET-CDC20 2.4CEN4*(2.4 kb right of *CEN4)::tetOs tetR-GFP SPC42-tdTomato ctf19-2A::HIS3*	This study
AM22226	*MATa 3yGFP-DBF4-13Myc::KanMX6*	This study
AM22230	*MATa 3yGFP-DBF4-FRB::KanMX6*	This study
AM22360	*MATa tor1-1 fpr1::NAT DBF4-FRB::KanMX ctf19-2A-FKBP12::TRP1 SCC2-6HIS-3FLAG::KanMX*	This study
AM22481	*MATa pMET-CDC20 2.4CEN4*(2.4 kb right of *CEN4*)*::tetOs tetR-GFP SPC42-tdTomato ctf19-2D::HIS3*	This study
AM22484	*MATa pMET-CDC20 2.4CEN4*(2.4 kb right of *CEN4*)*::tetOs tetR-GFP SPC42-tdTomato ctf19-2E::HIS3*	This study
AM22538	*MATa CDC7-6HIS-3FLAG::URA3 DBF4-13Myc::KanMX*	This study
AM22540	*MATa CDC7-6HIS-3FLAG::URA3 DBF4-FRB::KanMX*	This study
AM22672	*MATa tor1-1 fpr1::NAT DBF4-FRB::KanMX ctf19-2A-FKBP12::TRP1 SCC2-6HIS-3FLAG::KanMX ctf3Δ::KanMX*	This study
AM23268	*MATa CDC7-6HIS-3FLAG::URA3 ctf3Δ:: KanMX*	This study
AM23275	*MATa 3yGFP-DBF4 ctf3Δ:: KanMX*	This study
AM23456	*MATa SCC1-6HA ctf19-9A::HIS3*	This study
AM24195	*MATa CTF19-6HIS-3FLAG::natMX*	This study
AM24196	*MATa CTF19-6HIS-3FLAG::natMX ctf3Δ:: KanMX*	This study
SMH433	*MATa leu2Δ0::pGAL1-10::FLAG-cdc7-AS3-CBP-TEV-proA:DBF4::LEU2*	This Study
SMH425	*MATa CTF19-3FLAG::KanMX6*	This study
SMH426	*MATa ctf19-9A-3FLAG::KanMX6*	This study
SMH427	*MATa CTF19-3FLAG::KanMX6 dbf4-9myc::TRP1*	This study
SMH159	*MATa pMET-CDC20 1.8CEN15*(1.8 kb left of *CEN15*)*::lacO-256 lacI-*	Goshima and

	GFP	Yanagida, 2000
SMH412	*MATa pMET-CDC20 1.8CEN15*(1.8 kb left of *CEN15)::lacO-256 lacI-GFP CTF19-3FLAG::KanMX*	This study
SMH395	*MATa pMET-CDC20 1.8CEN15*(1.8 kb left of *CEN15)::lacO-256 lacI-GFP ctf19Δ::KanMX*	This study
SMH396	*MATa pMET-CDC20 1.8CEN15*(1.8 kb left of *CEN15)::lacO-256 lacI-GFP ctf19-9A-3FLAG::KanMX*	This study
SMH397	*MATa pMET-CDC20 1.8CEN15*(1.8 kb left of *CEN15)::lacO-256 lacI-GFP ctf19-3A-1-3FLAG::KanMX*	This study

Table S5. Related to STAR Methods – Yeast strains built in s288c

Strain Number	Genotype	Reference
SMH170	*MATa CDC7-CBP-TEV-2proA::HIS3*	Gaemmaghami et al., 2003
SMH372	*MATa CTF19-3FLAG::KanMX6*	This study
SMH378	*MATa ctf19-9A-3FLAG::KanMX6*	This study
SMH449	*MATa ctf19-2A-3FLAG::KanMX6*	This study
SMH450	*MATa ctf19-3A-2-3FLAG::KanMX6*	This study
SMH401	*MATa CTF19-3FLAG::KanMX6 chl4Δ::HIS3*	This study
SMH429	*MATa CTF19-3FLAG::KanMX6 chl4Δ::HIS3 leu2Δ0::pGAL1-10::6HIS-CDC7:DBF4::LEU2*	This study
SMH430	*MATa ctf19-9A-3FLAG::KanMX6 leu2Δ0::pGAL1-10::6HIS-CDC7:DBF4::LEU2*	This study
SMH404	*MATa CTF19-3FLAG::KanMX6 ctf3Δ::HIS3*	This study
SMH402	*MATa CTF19-3FLAG::KanMX6 dcc1Δ::HIS3*	This study
SMH403	*MATa CTF19-3FLAG::KanMX6 csm3Δ::HIS3*	This study
SMH477	*MATa CTF19-3FLAG::KanMX6 SLD7-GFP::HIS3 SPC110-mCherry::hphMX4*	This study
SMH480	*MATa ctf19-9A-3FLAG::KanMX6 SLD7-GFP::HIS3 SPC110-mCherry::hphMX4*	This study
SMH478	*MATa ctf19Δ::KanMX6 SLD7-GFP::HIS3 SPC110-mCherry::hphMX4*	This study
SMH473	*MATa CTF19-3FLAG::KanMX6 CDC7-GFP::HIS3 SPC110-mCherry::hphMX4*	This study
SMH476	*MATa ctf19-9A-3FLAG::KanMX6 CDC7-GFP::HIS3 SPC110-*	This study

	mCherry::hphMX4	
SMH474	*MATa ctf19Δ::KanMX6 CDC7-GFP::HIS3 SPC110-mCherry::hphMX4*	This study
SMH353	*MATa CTF19-3FLAG::KanMX6 SCC2-GFP::HIS3 MTW1-tdTomato::natMX*	This study
SMH361	*MATa ctf19Δ::KanMX6 SCC2-GFP::HIS3 MTW1-tdTomato::natMX*	This study
SMH354	*MATa ctf19-9A-3FLAG::KanMX6 SCC2-GFP::HIS3 MTW1-tdTomato::natMX*	This study
SMH355	*MATa ctf19-3A-1-3FLAG::KanMX6 SCC2-GFP::HIS3 MTW1-tdTomato::natMX*	This study
SMH451	*MATa ctf19-2A-3FLAG::KanMX6 SCC2-GFP::HIS3 MTW1-tdTomato::natMX*	This study
SMH452	*MATa ctf19-3A-2-3FLAG::KanMX6 SCC2-GFP::HIS3 MTW1-tdTomato::natMX*	This study
SMH459	*MATa ctf19-6A-3FLAG::KanMX6 SCC2-GFP::HIS3 MTW1-tdTomato::natMX*	This study
SMH460	*MATa ctf19-7A-3FLAG::KanMX6 SCC2-GFP::HIS3 MTW1-tdTomato::natMX*	This study
SMH416	*MATa csm3Δ::KanMX6 SCC2-GFP::HIS3 MTW1-tdTomato::natMX*	This study
SMH417	*MATa dcc1Δ::KanMX6 SCC2-GFP::HIS3 MTW1-tdTomato::natMX*	This study
